# Translating phenotypic prediction models from big to small anatomical MRI data using meta-matching

**DOI:** 10.1162/imag_a_00251

**Published:** 2024-08-01

**Authors:** Naren Wulan, Lijun An, Chen Zhang, Ru Kong, Pansheng Chen, Danilo Bzdok, Simon B. Eickhoff, Avram J. Holmes, B.T. Thomas Yeo

**Affiliations:** Centre for Sleep & Cognition & Centre for Translational Magnetic Resonance Research, Yong Loo Lin School of Medicine, National University of Singapore, Singapore, Singapore; Department of Electrical and Computer Engineering, National University of Singapore, Singapore, Singapore; N.1 Institute for Health, National University of Singapore, Singapore, Singapore; Department of Biomedical Engineering, McConnell Brain Imaging Centre (BIC), Montreal Neurological Institute (MNI), Faculty of Medicine, School of Computer Science, McGill University, Montreal QC, Canada; Mila – Quebec Artificial Intelligence Institute, Montreal, QC, Canada; Institute for Systems Neuroscience, Medical Faculty, Heinrich-Heine University Düsseldorf, Düsseldorf, Germany; Institute of Neuroscience and Medicine, Brain & Behavior (INM-7), Research Center Jülich, Jülich, Germany; Department of Psychiatry, Brain Health Institute, Rutgers University, Piscataway, NJ, United States; Integrative Sciences and Engineering Programme (ISEP), National University of Singapore, Singapore, Singapore; Martinos Center for Biomedical Imaging, Massachusetts General Hospital, Charlestown, MA, United States; Department of Medicine, Human Potential Translational Research Programme & Institute for Digital Medicine (WisDM), Yong Loo Lin School of Medicine, National University of Singapore, Singapore, Singapore

**Keywords:** structural MRI, transfer learning, meta-matching, phenotypic prediction

## Abstract

Individualized phenotypic prediction based on structural magnetic resonance imaging (MRI) is an important goal in neuroscience. Prediction performance increases with larger samples, but small-scale datasets with fewer than 200 participants are often unavoidable. We have previously proposed a “meta-matching” framework to translate models trained from large datasets to improve the prediction of new unseen phenotypes in small collection efforts. Meta-matching exploits correlations between phenotypes, yielding large improvement over classical machine learning when applied to prediction models using resting-state functional connectivity as input features. Here, we adapt the two best performing meta-matching variants (“meta-matching finetune” and “meta-matching stacking”) from our previous study to work with T1-weighted MRI data by changing the base neural network architecture to a 3D convolution neural network. We compare the two meta-matching variants with elastic net and classical transfer learning using the UK Biobank (N = 36,461), the Human Connectome Project Young Adults (HCP-YA) dataset (N = 1,017), and the HCP-Aging dataset (N = 656). We find that meta-matching outperforms elastic net and classical transfer learning by a large margin, both when translating models within the same dataset and when translating models across datasets with different MRI scanners, acquisition protocols, and demographics. For example, when translating a UK Biobank model to 100 HCP-YA participants, meta-matching finetune yielded a 136% improvement in variance explained over transfer learning, with an average absolute gain of 2.6% (minimum = –0.9%, maximum = 17.6%) across 35 phenotypes. Overall, our results highlight the versatility of the meta-matching framework.

## 1 Introduction

An important goal in systems neuroscience is to understand how variation in brain structure relates to individual differences in behavior (Genon et al., 2022). Structural T1-weighted magnetic resonance imaging (MRI) is a noninvasive technique for examining the anatomy of the human brain, providing high contrast between gray and white matter (Gifford et al., 2020). Structural MRI is widely used to predict behavioral traits, clinical symptoms, and diagnostic categories in both healthy individuals and individuals with neuropsychiatric disorders (Arbabshirani et al., 2017;Bhagwat et al., 2019;Cohen et al., 2021;Ooi et al., 2022;Sabuncu et al., 2015). However, most prediction studies use datasets with fewer than a few hundred participants, leading to low reproducibility and inflated performance (Arbabshirani et al., 2017;Bzdok & Meyer-Lindenberg, 2018;Marek et al., 2022;Masouleh et al., 2019;Poldrack et al., 2020). Studies have shown that prediction performance increases with larger sample sizes (Chu et al., 2012;Cui & Gong, 2018;He et al., 2020;Schulz et al., 2020), but for investigations of certain clinical populations or focused neuroscience inquiries, small-scale datasets remain unavoidable. Here, to address this fundamental issue, we seek to establish a framework to translate prediction models from large-scale datasets to predict new nonbrain-imaging phenotypes in small-scale datasets based on anatomical T1-weighted images.

More specifically, given a large-scale anatomical MRI dataset (N > 10,000) with multiple phenotypes, we seek to translate models trained from the large dataset to new unseen phenotypes in a small independent dataset (N ≤ 200). In machine learning, this problem is often referred to as meta-learning, lifelong learning, learning-to-learn, or few-shot learning (Andrychowicz et al., 2016;Fei-Fei et al., 2006;C. Finn et al., 2017;Ravi & Larochelle, 2016;Vanschoren, 2019), and is closely related to transfer learning (Hospedales et al., 2021;Weiss et al., 2016). Broadly speaking, meta-learning and transfer learning methods usually train a model on abundant data on a related problem, called the source dataset, and seek to translate knowledge learned from the large-scale dataset to the small dataset, called the target dataset. During the translation, a subset of the target dataset is typically used to adapt the pretrained model to the new sample. One distinction between meta-learning and transfer learning is that in transfer learning, the prediction problem in the target dataset can be same (Aderghal et al., 2018;Ghafoorian et al., 2017;Wee et al., 2019) or different (Dawud et al., 2019;Mehmood et al., 2021;Talo et al., 2019) from the source dataset. On the other hand, meta-learning always involves the translation of the prediction model to perform a*new*prediction problem in the target dataset—providing the imaging neuroscience community with a versatile modeling framework that, once established, can be applied to a diversity of research goals.

In our previous study (He et al., 2022), we developed a simple “meta-matching” approach to translate prediction models from large datasets to improve the prediction of new phenotypes in small datasets. Meta-matching is grounded in the observation that many phenotypes are correlated, as demonstrated by previous studies identifying a small number of factors linking brain-imaging data to various nonbrain-imaging traits such as cognition, mental health, demographics, and other health attributes (Kebets et al., 2019;Miller et al., 2016;Smith et al., 2015;Xia et al., 2018). As a result, a phenotype X in a smaller-scale study is likely correlated, sharing a latent relationship, with a phenotype Y present in a larger population dataset. Therefore, a model trained to predict phenotype Y from the larger dataset might be predisposed to features useful for predicting phenotype X. Consequently, the predictive model of Y can be more effectively translated to predict phenotype X in the smaller study. As a demonstration of meta-matching (He et al., 2022), we trained a simple fully connected feedforward neural network to predict 67 nonbrain-imaging phenotypes from resting-state functional connectivity (RSFC) in the UK Biobank. The neural network was then translated using meta-matching to predict nonbrain-imaging phenotypes in the Human Connectome Project Young Adult (HCP-YA) dataset, yielding large improvements in prediction accuracies over classical kernel ridge regression (without meta-learning or transfer learning).

In the current study, we investigated whether the two best performing meta-matching variants (“meta-matching finetune” and “meta-matching stacking”) from our previous study (He et al., 2022) can be adapted to work with T1 MRI data. More specifically, given the different modalities (RSFC versus T1), the base neural network architecture was changed from a fully connected feedforward neural network to the simple fully convolutional network (SFCN;Peng et al., 2021). The SFCN was chosen because of its simplicity and top performance in the Predictive Analysis Challenge 2019 of brain age prediction (Peng et al., 2021). We compared the two meta-matching variants with classical elastic net and classical transfer learning using the UK Biobank (Miller et al., 2016;Sudlow et al., 2015), the Human Connectome Project Young Adults (HCP-YA) dataset (Van Essen et al., 2013), and the HCP-Aging dataset (Bookheimer et al., 2019;Harms et al., 2018).

It is worth mentioning that it is not obvious that meta-matching will confer great benefits in anatomical MRI, compared with RSFC (He et al., 2022). The reason is that RSFC-based prediction typically utilizes high-dimensional features derived from N × N RSFC matrices, where N is the number of brain parcels (or independent component analysis components). On the other hand, T1-based prediction can utilize low-dimensional N × 1 volumetric and/or thickness measures. Therefore, classical machine learning techniques (e.g., elastic net) might work really well in the small sample regime (≤200 participants). Nevertheless, we found that meta-matching significantly outperformed classical elastic net and transfer learning, highlighting the versatility of the meta-matching framework.

## 2 Methods

### 2.1 Datasets and preprocessing

In this section, we describe the datasets and preprocessing used in the current study.Table 1summarizes the demographics and acquisition parameters of the three datasets we considered. We will evaluate meta-matching based on prediction accuracy when translating prediction models within the same dataset (UK Biobank), as well as across datasets, i.e., from the UK Biobank to the HCP-YA and HCP-Aging datasets. The very different age ranges between the HCP-YA and UK Biobank served as a strong test of the generalizability of meta-matching. All data collection and analysis procedures were approved by the respective institutional review boards (IRBs), including the National University of Singapore IRB for the analysis presented in this paper.

**Table 1.** Summary of demographics and acquisition parameters of the three datasets used in the current study.

	Age	Sex (M/F)	Scanner(s)	Resolution
UK Biobank	45-82	53%/47%	Siemens Skyra 3T scanner	1 mm
HCP-YA	22-35	47%/53%	Customized Skyra 3T scanner	0.7 mm
HCP-aging	36-100	44%/56%	Siemens Prisma 3T scanner	0.8 mm

#### 2.1.1 UK Biobank

The UK Biobank (UKBB) dataset is a large-scale epidemiology study of over 500,000 adults from the United Kingdom (Alfaro-Almagro et al., 2018). The volunteers were recruited between 2006 and 2010 from 22 centers across the United Kingdom. Participants were asked to answer a variety of questionnaires about different aspects of health and lifestyle. In addition, a range of physiological measurements was also collected. We considered the same set of 36,848 participants and 67 nonbrain-imaging phenotypes (referred to as phenotypes henceforth;Table S1) from our previous study (He et al., 2022).

As part of the UK Biobank pipeline (Alfaro-Almagro et al., 2018), FreeSurfer recon-all was used to derive thickness and volume measures with the Desikan-Killiany-Tourville (DKT40) cortical atlas (Klein & Tourville, 2012) and subcortical segmentation (Fischl et al., 2002). We considered the subset of regions present in most participants, yielding 164 morphometric measures, comprising intracranial volume (ICV) and thickness measures of 62 cortical regions, as well as volumes of 62 cortical regions and 39 subcortical gray-matter regions (Tables S4 and S5). After excluding participants who have dropped out from our previous study (He et al., 2022) and excluding participants without all 164 morphometric measures, we ended up with 36,461 participants. As a baseline, these 164 measures will be utilized by the elastic net algorithm for phenotypic prediction (seeSection 2.3).

Furthermore, we used FMRIB’s Linear Image Registration Tool (FLIRT) to transform the bias-field-corrected version of the brain-extracted T1 (from the UK Biobank provided preprocessing outputs) to the FSL MNI152 standard-space T1 template with 1 mm resolution (Jenkinson et al., 2002;Jenkinson & Smith, 2001). Each T1 image was cropped to dimensions 160 x 192 x 160, and then divided by the mean value within each image followingPeng et al. (2021). The normalized T1 images will be used by a convolutional neural network for phenotypic prediction (Section 2.3).Table 2summarizes the preprocessing steps for the 3D T1 images for the UK Biobank, HCP-YA, and HCP-Aging datasets.

**Table 2.** Summary of preprocessing steps for the 3D T1 images for the UK Biobank, HCP-YA, and HCP-Aging datasets.

Step #	UK Biobank	HCP pipeline
1	Gradient distortion correction	Gradient distortion correction
2	Brain extraction (BET) and cut down FOV.	Averaging of the same files (if multiple scans of the same modality exist)
3	Registration to standard space (linear) (FLIRT)	AC-PC alignment
4	Registration to standard space (nonlinear) (FNIRT)	Image FNIRT-based brain extraction
5	Create brain mask from MNI152_T1_1 mm, then do brain extraction	T2w to T1w image registration
6	Defacing	Readout distortion correction
7	Bias correction (the bias field is estimated using FAST)	Bias field correction
8	Tissue and subcortical structure segmentation	Linear and nonlinear atlas registration to MNI152

#### 2.1.2 HCP young adult (HCP-YA) dataset

We utilized the Human Connectome Project Young Adult (HCP-YA) dataset (Van Essen et al., 2013), which included healthy young adults. We considered 1,019 participants and 35 nonbrain-imaging phenotypes, consistent with our previous study (He et al., 2022). The phenotypes are found inTable S2.

FreeSurfer recon-all from the HCP pipeline was used to derive thickness and volume measures with the DKT40 cortical atlas (Klein & Tourville, 2012) and ASEG subcortical segmentation (Fischl et al., 2002). We considered the subset of regions present in most participants, yielding 166 morphometric measures, comprising intracranial volume (ICV) and thickness measures of 62 cortical regions, as well as volumes of 62 cortical regions and 41 subcortical gray-matter regions (Tables S4 and S5). We note that the difference in the number of morphometric measures between the UK Biobank and HCP-YA datasets (164 vs. 166) arose because the 5th-Ventricle and non-WM-hypointensities were missing in most participants from the UK Biobank dataset. As a baseline, these 166 measures will be utilized by the elastic net algorithm for phenotypic prediction (seeSection 2.3).

Moreover, we considered T1 images of 0.7 mm resolution which had been transformed to FSL MNI152 space by FLIRT from the HCP PreFreesurfer pipeline (Glasser et al., 2013), which included gradient distortion correction, brain extraction, and readout distortion correction. We noted the files of two participants were missing in the HCP filesystem, so we ended up with 1,017 participants. Each T1 image was downsampled to 1 mm, cropped to dimensions 160 x 192 x 160, and then divided by the mean value within each image followingPeng et al. (2021). The processed T1 images will be used by a convolutional neural network for phenotypic prediction (Section 2.3).

#### 2.1.3 HCP-aging dataset

Besides the HCP-YA dataset, we also used the Human Connectome Project Aging (HCP-Aging) dataset (Bookheimer et al., 2019;Harms et al., 2018) consisting of healthy participants. We manually selected commonly used nonbrain-imaging phenotypic measures across cognition, emotion, motor, sensor, and life experience, resulting in 45 phenotypes (Table S3). By only considering participants with at least 90% of the phenotypes, we ended up with 656 participants (out of 725 participants). Similar to the HCP-YA dataset, we used the same 166 morphometric measures generated by the FreeSurfer recon-all procedure from the HCP pipeline. Moreover, we considered T1 images of 0.8 mm resolution, which had been transformed to FSL MNI152 space by FLIRT from the HCP PreFreesurfer pipeline (Glasser et al., 2013), which included gradient distortion correction, brain extraction, and readout distortion correction. Each T1 image was downsampled to 1 mm, cropped to dimensions 160 x 192 x 160, and then divided by the mean value within each image followingPeng et al. (2021). The processed T1 images will be used by a convolutional neural network for phenotypic prediction (Section 2.3).

### 2.2 Data split for different analyses

We performed two sets of analyses. First, we benchmarked meta-matching within the UK Biobank. Second, we translated predictive models from the UK Biobank to the HCP-YA and HCP-Aging datasets.

#### 2.2.1 Data split within the UK Biobank

For the UK Biobank analysis, we considered 36,461 participants with T1 structural MRI and 67 phenotypes. As illustrated inFigure 1, we randomly split the data into a meta-training set comprising 26,573 participants with 33 phenotypes, as well as a meta-test set comprising 9,888 participants with 34 phenotypes. There was no overlap between the participants and phenotypes across the meta-training set and meta-test set.

**Fig. 1.**
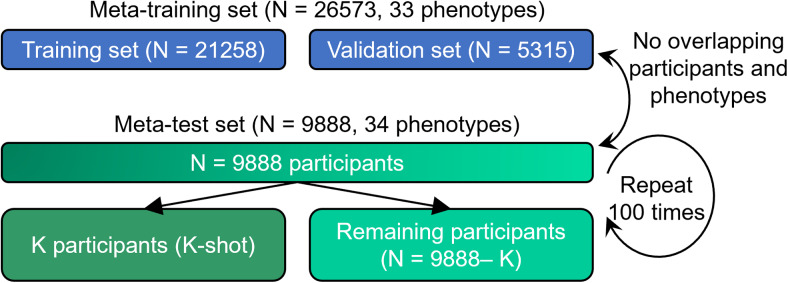
Data split scheme for within-UK Biobank analysis. The UK Biobank dataset was divided into a meta-training set comprising 26,573 participants and 33 phenotypes, as well as a meta-test set comprising 9,888 participants and 34 other phenotypes. There was no participant or phenotype overlap between the meta-training and meta-test sets. The meta-test set was, in turn, split into K participants (K = 10, 20, 50, 100, and 200) and remaining 9,888 − K participants. The group of K participants mimicked studies with traditionally common sample sizes. Various trained models from the meta-training set were translated to the meta-test set using the K participants. The models were then evaluated using the remaining N – K participants. This random split was repeated 100 times for robustness.

We further randomly split the meta-training set into a training set with 21,258 participants (80% of 26,573 participants) and a validation set with 5,315 participants (20% of 26,573 participants). The validation set was used for tuning hyperparameters of the predictive models.

For the meta-test set, we randomly split 9,888 participants into K participants (K-shot) and 9,888− K participants, where K had a value of 10, 20, 50, 100, and 200. The group of K participants mimicked traditional small-N studies. Various trained models from the meta-training set were translated to the meta-test set using the K participants. The models were then evaluated using the remaining N – K participants. Each random K-shot split was repeated 100 times to ensure stability.

#### 2.2.2 Data split scheme for cross-dataset analyses

To translate predictive models from the UK Biobank to other datasets, we considered the HCP-YA and HCP-Aging datasets. As illustrated inFigure 2, the meta-training set comprised all 36,461 participants with all 67 phenotypes from the UK Biobank dataset. The first meta-test set consisted of 1,017 participants with 35 phenotypes from the HCP-YA dataset. The second meta-test set consisted of 656 participants with 45 phenotypes from the HCP-Aging dataset. There was no overlap between the participants and phenotypes across the meta-training and meta-test sets because they were from totally different datasets. For the meta-training set, we further randomly split it into a training set with 29,169 participants (80% of 36,461 participants) and a validation set with 7,292 participants (20% of 36,461 participants). The validation set was used for tuning hyperparameters of the predictive models.

**Fig. 2.**
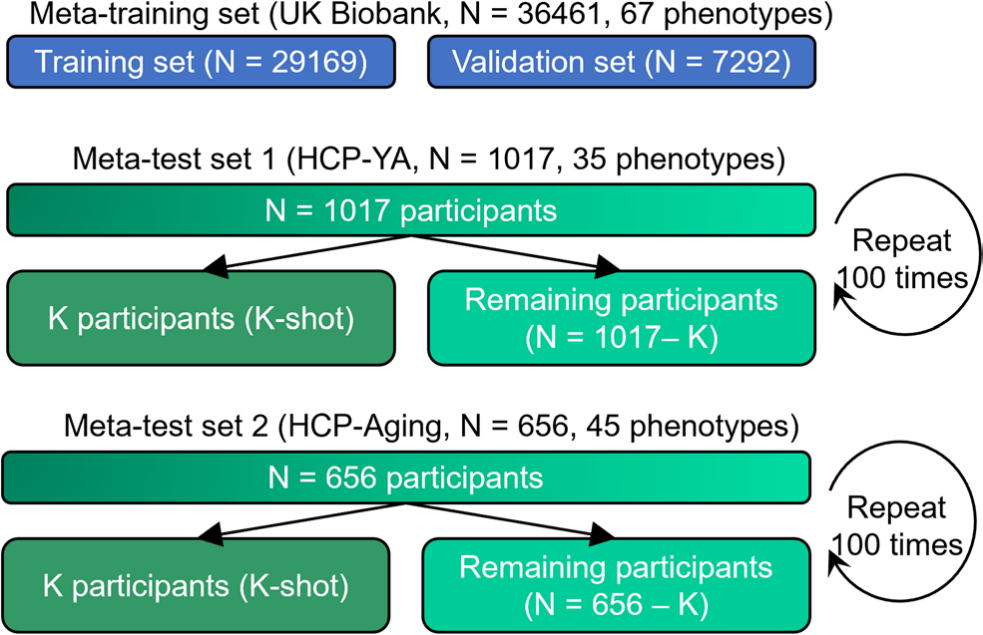
Data split scheme for cross-dataset analysis. The meta-training set comprised 36,461 UK Biobank participants and 67 phenotypes. The first meta-test set comprised 1,017 HCP-YA participants and 35 phenotypes. The second meta-test set comprised 656 HCP-Aging participants and 45 phenotypes. Each meta-test was, in turn, split into K participants (K = 10, 20, 50, 100, and 200) and the remaining participants. The group of K participants mimicked studies with traditionally common sample sizes. Various trained models from the meta-training set were translated to the meta-test set using the K participants. The models were then evaluated using the remaining N – K participants. This random split was repeated 100 times for robustness.

For the HCP-YA dataset, we randomly split 1,017 participants into K participants (K-shot) and 1,017− K participants, where K had a value of 10, 20, 50, 100, and 200. Various trained models from the meta-training set were translated to the meta-test set using the K participants. The models were then evaluated using the remaining N – K participants. Each random K-shot split was repeated 100 times to ensure stability. The same procedure was applied to the HCP-Aging dataset.

### 2.3 Predictive models

**Fig. 3.**
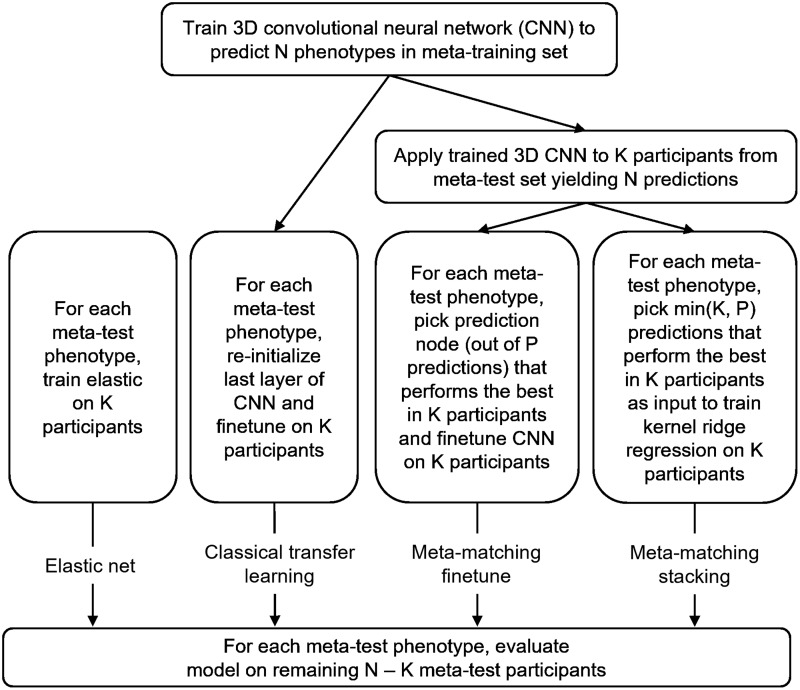
Overview of different approaches. We considered two baselines: elastic net and classical transfer learning. We proposed two meta-matching variants: meta-matching finetune and meta-matching stacking.

Figure 3provides an overview of the different approaches we will compare. Across all approaches, we z-normalize each nonimaging phenotype to have zero mean and unit variance across participants. More specifically, in the case of the meta-training set, the mean and standard deviation were computed using all the participants to apply the z-normalization. In the case of the meta-test set, for each phenotype, the mean and standard deviation were computed from the K participants and subsequently carried over to the full meta-test set comprising the K participants and the remaining N – K test participants.

Following our previous study (He et al., 2022), statistical difference between algorithms was evaluated using a bootstrapping approach (more details inSupplementary Methods S2). More specifically, we will compare both meta-matching variants (Sections 2.3.3and2.3.4) with the two baselines (Sections 2.3.1and2.3.2). Multiple comparisons were corrected using a false discovery rate (FDR) of q < 0.05. FDR was applied to all K-shots, across all comparisons and both evaluation metrics: Pearson’s correlation and coefficient of determination (COD). Formula for COD (Feilong et al., 2021) is found inSupplementary Methods S3.

#### 2.3.1 Baseline 1: elastic net

As a baseline, we used thickness and volumetric measures as input features to predict individuals’ phenotypes using elastic net (Fig. 3). Elastic net is a linear regression model with an L1 lasso and L2 ridge regularization terms (Zou & Hastie, 2005). Here, we chose elastic net as a baseline because previous studies have suggested that the elastic net yielded strong prediction performance in phenotypic prediction for brain MRI data (Ooi et al., 2022;Peng et al., 2021;Pervaiz et al., 2020).

As a reminder, we had 62 cortical regions and 39 subcortical gray-matter regions in the UK Biobank (Section 2.1.1). The cortical regions yielded cortical thickness and volumetric measures, while the subcortical regions yielded volumetric measures. Together with the intracranial volume, this results in 1 + 62 x 2 + 39 = 164 morphometric features that were fed into the elastic net. In the case of the HCP-YA and HCP-Aging datasets, we had 62 cortical regions and 41 subcortical gray-matter regions (Sections 2.1.2and2.1.3), yielding 1 + 62 x 2 + 41 = 166 morphometric features.

We note that the range of values is very different for volumetric and thickness measures. Therefore, given K participants from the meta-test set, the morphometric (volumetric and thickness) measures were z-normalized based on the mean and standard deviation computed from the K participants. We note that the morphometric measures of the N – K participants were also z-normalized using the mean and standard deviation computed from the K participants. The z-normalized morphometric measures were used as input to train the elastic net model on the K participants.

More specifically, we performed fivefold cross-validation on the K participants with different combinations of the hyperparameters λ_1_and λ_2_(which controlled the strength of the L1 and L2 regularizations). We used COD to evaluate prediction performance to choose the best hyperparameters for λ_1_and λ_2_across the fivefold cross-validation.

The best hyperparameters λ_1_and λ_2_were then used to train the elastic net model using all K participants. The trained elastic net model was then applied to the remaining N – K test participants in the meta-test set. Pearson’s correlation and the COD were used to evaluate prediction performance. This procedure was repeated for each of the 100 random splits.

#### 2.3.2 Baseline 2: classical transfer learning

To perform classical transfer learning, we first trained a simple fully convolutional network (SFCN) introduced byPeng et al. (2021)in the meta-training set to jointly predict all the available meta-training phenotypes.

The input to the SFCN is the mean-normalized T1 image affine transformed to MNI152 standard space (Section 2.1). The SFCN’s convolutional neural network (CNN) architecture was based on VGG (Simonyan & Zisserman, 2014) and used a fully convolutional structure (Long et al., 2015). We chose the SFCN given its simplicity and top performance in the Predictive Analysis Challenge 2019 of brain age prediction (Peng et al., 2021). In the original study (Peng et al., 2021), the last layer comprised 40 nodes that represented the predicted probability of the age interval that a participant’s age falls into. Here, we modified the last layer to predict P phenotypes simultaneously. P is equal to 33 in the within-UK Biobank analysis (Fig. 1) and P is equal to 67 in the cross-dataset analysis (Fig. 2).

**Fig. 4.**
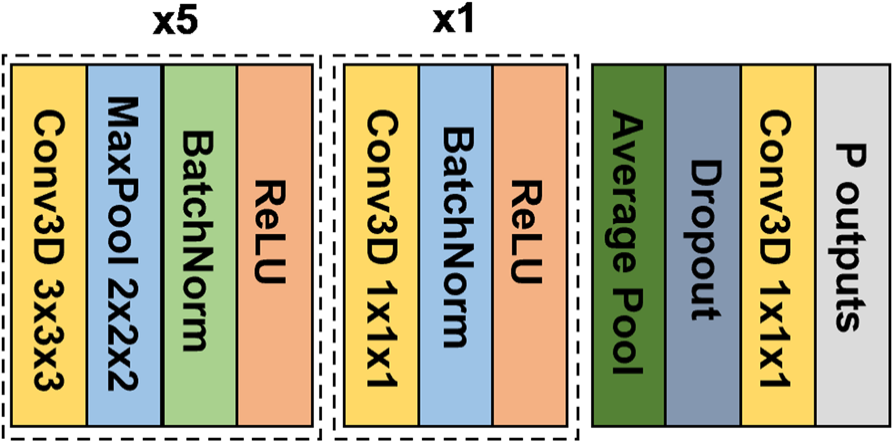
Network architecture of the Simple Fully Convolutional Neural Network (SFCN) model (Peng et al., 2021) adapted to the current study. In the original study (Peng et al., 2021), the last layer comprised 40 nodes that represented the predicted probability of the age interval that a participant’s age falls into. Here, we modified the last layer to predict P phenotypes simultaneously.

Figure 4shows the overall network architecture. The 3D CNN consisted of several convolutional blocks for feature extraction. Each feature extraction block (except the last block) consisted of a 3D convolutional layer, a batch normalization layer, a max pooling layer, and a ReLU activation layer. The last block was similar to the previous blocks but without the max pooling layer. The feature maps from the last block were fed into an average pooling layer (green inFig. 4).

Since the elastic net utilized ICV as one of the features, while affine registration of T1 to MNI152 space removed this information, for the comparison to be comparable, we concatenated z-normalized ICV with the outputs of the average pooling layer. More specifically, for both meta-training and meta-test sets, ICV of each participant was z-normalized using the mean and standard deviation computed from the participants of the training set within the meta-training set. The concatenated features were then fed into a dropout layer and then went through a 3D convolution layer with 1 x 1 x 1 kernel size to produce the final outputs.

The hyperparameters of the CNN were empirically determined based on the meta-training set from the within-UK Biobank analysis (Fig. 1A). Both within-UK Biobank and cross-dataset analyses used the same set of hyperparameters. More details about model architecture and hyperparameters (e.g., the number of blocks, number of channels per block, and kernel size per block) can be found inSupplementary Methods S1.

The same transfer learning procedure was used for both within-UK Biobank analysis and cross-dataset analysis (Fig. 3). The only difference is that the within-UK Biobank analysis used a CNN model trained on 26,573 participants and 33 phenotypes, while the cross-dataset analysis used a CNN model trained on 36,461 participants and 67 phenotypes.

To perform transfer learning, we first replaced the last layer of the 3D CNN model (trained on the meta-training set) with a new convolutional layer with 1 x 1 x 1 kernel size and one output node. The new convolutional layer was initialized with random weights. For each meta-test phenotype, the last two layers of the CNN model were then finetuned on K participants in the meta-test set, while the weights of the remaining layers were frozen.

The optimal learning rate was determined using grid search and fivefold cross-validation on the K participants. After choosing the optimal learning rate, it was then used to train a final model using all K participants. For both the fivefold cross-validation and the final round of finetuning, the maximum finetuning epochs were set to be 50 with 80% of K participants used for training and 20% used to evaluate validation loss for early stopping, to reduce the possibility of overfitting. This final trained model was evaluated in the remaining N – K participants in the meta-test set. Pearson’s correlation and COD were used to evaluate the prediction performance. This procedure was repeated for each of the 100 random splits.

#### 2.3.3 Meta-matching finetune

As an alternative to transfer learning, we considered the “meta-matching finetune” approach (Fig. 3) introduced in our previous study (He et al., 2022). To explain the meta-matching finetune procedure in the current study, we will focus on the experimental setup for the within-UK Biobank analysis.

Recall fromSection 2.3.2that we have trained a 3D CNN model to predict 33 phenotypes in the meta-training set from the UK Biobank. Given K participants from the meta-test set, we applied the CNN yielding 33 predictions. For each meta-test phenotype (out of 34 phenotypes), we calculated the accuracy (COD) with each of the 33 predictions for the K participants. The output node of the CNN model with the best COD was chosen, while the remaining 32 nodes were removed. The last two layers of the CNN model were finetuned using the K participants, while the weights of the remaining layers were frozen.

Therefore, the difference between meta-matching finetune and classical transfer learning (Section 2.3.2) is the initialization of the last layer. Classical transfer learning randomly initialized the last layer, while meta-matching finetune initialized the last layer by choosing the “closest” phenotypic prediction model from the meta-training set.

The optimal learning rate for finetuning was determined using grid search and fivefold cross-validation on the K participants. After choosing the optimal learning rate, it was then used to train a final model using all K participants. For both the fivefold cross-validation and the final round of finetuning, the maximum finetuning epochs were set to be 50 with 80% of K participants used for training and 20% used to evaluate validation loss for early stopping, to reduce the possibility of overfitting.

This final trained model was evaluated in the remaining N – K participants in the meta-test set. Pearson’s correlation and COD were used to evaluate the prediction performance. This procedure was repeated for each of the 100 random splits. The same procedure was used for both the within-UK Biobank analysis and cross-dataset analysis (Fig. 3). The only difference was that the within-UK Biobank analysis used a CNN model trained on 26,573 participants and 33 phenotypes, while the cross-dataset analysis used a CNN model trained on 36,461 participants and 67 phenotypes.

#### 2.3.4 Meta-matching stacking

We also considered the meta-matching stacking approach (Fig. 3) introduced in our previous study (He et al., 2022). To explain the meta-matching stacking procedure in the current study, we will again focus on the experimental setup for the within-UK Biobank analysis.

Recall fromSection 2.3.2that we have trained a 3D CNN model to predict 33 phenotypes in the meta-training set from the UK Biobank. Given K participants from the meta-test set, we applied the CNN yielding 33 predictions. For each meta-test phenotype (out of 34 phenotypes), we calculated the accuracy (COD) with each of the 33 predictions for the K participants, and selected the top M predictions. The value of M was set to be the minimum of K and 33 to reduce overfitting. For example, when K = 20, then M was set to be 20. When K = 50, then M was set to be 33.

A stacking procedure was then performed (Breiman, 1996;Wolpert, 1992), in which a kernel ridge regression (KRR) model was trained on K participants using the M predictions as input to predict the meta-test phenotype. Similar to our previous study (He et al., 2022), we used the correlation kernel. The hyperparameter λ was tuned using grid search and fivefold cross-validation on the K participants. The optimal λ was then used to train a final KRR model using all K participants.

The trained KRR model was then applied to the remaining N – K participants in the meta-test set. Pearson’s correlation and the COD were used to evaluate the prediction performance. This procedure was repeated for each of the 100 random splits.

### 2.4 Deep neural network implementation

The deep neural network was implemented using PyTorch (Paszke et al., 2017) and computed on NVIDIA RTX 3090 GPUs with CUDA 11.0. More details are found inSupplementary Methods S1.

### 2.5 Model interpretation

Future studies using our pretrained models would have to interpret the resulting meta-matching models. Therefore, to illustrate how meta-matching models can be interpreted, similar to our previous study (He et al., 2022), we utilized the Haufe transform (Haufe et al., 2014) to interpret the meta-matching stacking prediction of the Rey Auditory Verbal Learning Test (RAVLT) score and Montreal Cognitive Assessment (MOCA) in the HCP-Aging dataset for K = 100 participants.

For a predictive model with T1 structural MRI as input and phenotype as output, Haufe transform produces a feature importance value for each voxel. A positive (or negative) predictive feature value implied that higher T1 intensity was related to predicting greater (or lower) phenotypic score.

More specifically, for each phenotype, Haufe transform was calculated as the covariance between the phenotype’s prediction based on the meta-matching stacking model and the intensity value of each T1 voxel (across the 100 participants), yielding a 3D volume. The 3D volumes were averaged across the 100 random sampling of 100 participants, and were then visualized in MNI152 space.

We chose cognitive measures from the HCP-Aging dataset because there is a vast literature studying the relationships between aging and brain structures. As such, we expected that smaller hippocampal volume and larger ventricular size were predictive of worse cognition. However, because the model was trained on T1 intensities, careful inference is necessary to link the feature importance values of T1 intensities with more neuroanatomically grounded interpretations, e.g., how interindividual variation in hippocampal volume predicts cognition.

### 2.6 Computational costs

Meta-matching training comprised two stages. The first stage was to train the 3D CNN model on the meta-training set. The second stage adapted the model to K participants from a new target dataset. Finally, the adapted model was tested on the remaining N – K participants in the meta-test set, where N was the total number of participants in the meta-test set.

In our study, the 3D CNN model was trained on the meta-training set, using a single GPU (RTX3090 with 24 GB GPU memory). The whole training procedure took around 5 days. For the second stage involving K meta-test participants, meta-matching stacking required a single forward pass through the 3D CNN model, followed by training a KRR model on the K participants for each target phenotype. For each random sampling of K participants, for each value of K and for each phenotype, the training time was around 0.2 seconds (inclusive of the fivefold cross-validation to select the best hyperparameter). Evaluating the trained meta-matching stacking model on the N – K participants was a lot faster. For each value of K and each phenotype, evaluation for each random sampling of K participants required around 0.0007 seconds per 100 test participants.

On the other hand, the computational costs for meta-matching finetune and classical transfer learning were about the same, but a lot more than meta-matching stacking. For each random sampling of K participants and for each phenotype, the training time for meta-matching finetune (or transfer learning) was about 5 seconds for K = 10 or 20, and about 30 seconds for K = 50, 100, or 200 (inclusive of the fivefold cross-validation to select the best hyperparameter). Evaluation in the N – K participants was a lot faster. For each value of K and each phenotype, evaluation for each random sampling of K participants required around 0.0193 seconds per 100 test participants.

Because of the expensive training costs for meta-matching finetune and classical transfer learning, the bootstrapping procedure to obtain p values could not be applied to meta-matching finetune and classical transfer learning. With one GPU, the entire training procedure of the within-UK Biobank, HCP-YA, and HCP-Aging analyses with 1000 bootstraps would require around (5 seconds × 2 + 30 seconds × 3) × 1000 bootstraps × 114 phenotypes = 11,400,000 seconds or 132 days. Doing this for both meta-matching finetune and classical transfer learning would then require 264 days. On our computing facility, we might on average be able to utilize four GPUs (depending on load), so the total run time would be 264 /4 = 66 days.

In the case of elastic net, with 164 (or 166) morphometric features, for each random sampling of K participants and for each phenotype, the training time was about 3 seconds for K = 10 or 20, and was about 6 seconds for K = 50, 100, or 200. For each value of K and each phenotype, evaluation for each random sampling of K participants required around 0.0028 seconds per 100 test participants.

## 3 Results

### 3.1 Meta-matching outperforms elastic net and transfer learning within the UK Biobank

Four approaches (elastic net, classical transfer learning, meta-matching finetune, and meta-matching stacking) were applied to the UK Biobank to predict 34 meta-test phenotypes. The models were trained or adapted based on K participants and then evaluated on the remaining 9,888 – K participants (Fig. 1).

**Fig. 5.**
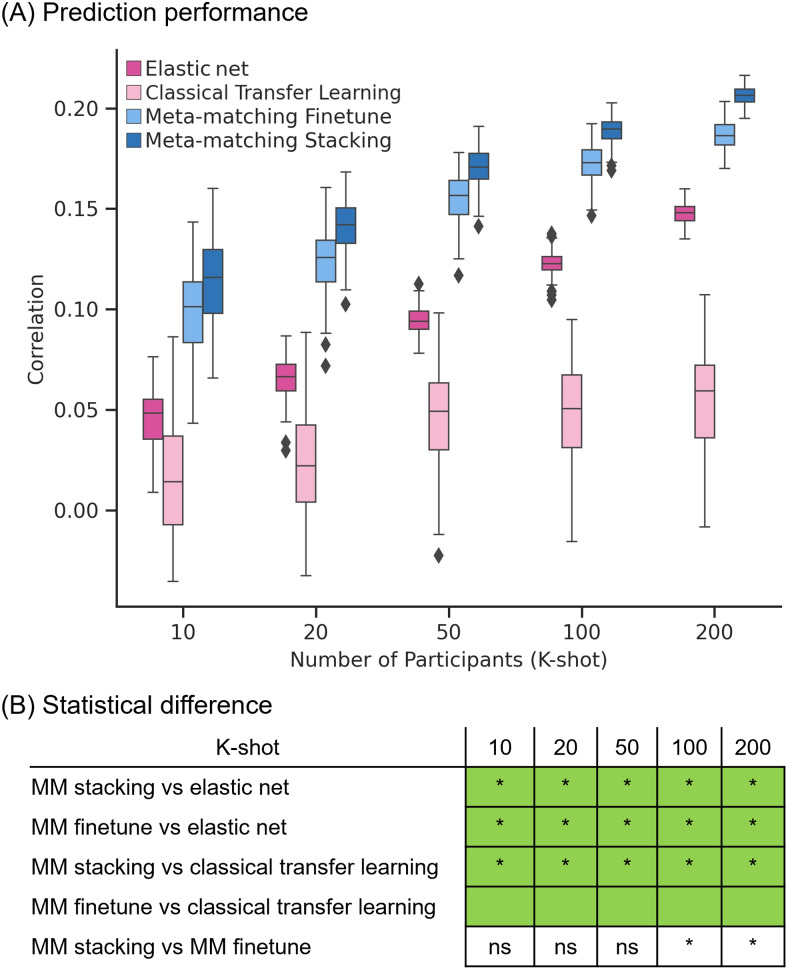
Meta-matching compared favorably with elastic net and direct transfer learning in terms of Pearson’s correlation within the UK Biobank. (A) Phenotypic prediction performance (Pearson’s correlation) averaged across 34 meta-test phenotypes in the UK Biobank. X-axis represents the number of participants in the meta-test set of the UK Biobank used to train an elastic net or adapt the pretrained model from the meta-training set of the UK Biobank. Each boxplot shows the distribution of performance over 100 repetitions of sampling K participants. (B) Statistical difference between the prediction performance (Pearson’s correlation) of baseline methods and meta-matching algorithms. p Values were calculated based on a two-sided bootstrapping test. “*” indicates statistical significance after multiple comparisons correction (FDR q < 0.05). “ns” indicates that statistical test did not survive FDR correction. We note that there was no statistical test between meta-matching finetune and classical transfer learning because the bootstrapping procedure was too expensive for the two methods. Colors indicate effect sizes of differences (Cohen’s D) between approaches. Light green indicates effect size ≥ 0.8. Dark green indicates 0 ≤ effect size < 0.8. Dark pink indicates −0.8 < effect size < 0. Light pink indicates effect size ≤ −0.8. There is no color for the comparison between meta-matching finetune and stacking since they are both our proposed methods.

Figures 5Aand6Ashow the Pearson’s correlation and COD, respectively, averaged across all 34 meta-test phenotypes. Each boxplot represents 100 random samplings of K participants.Figures 5Band6Bshow the outcomes of the statistical tests obtained by a bootstrapping procedure (Supplementary Methods S2 and Figure S1). The actual p values are reported inTable S6. Colors indicate effect sizes of differences (Cohen’s D) between approaches.

**Fig. 6.**
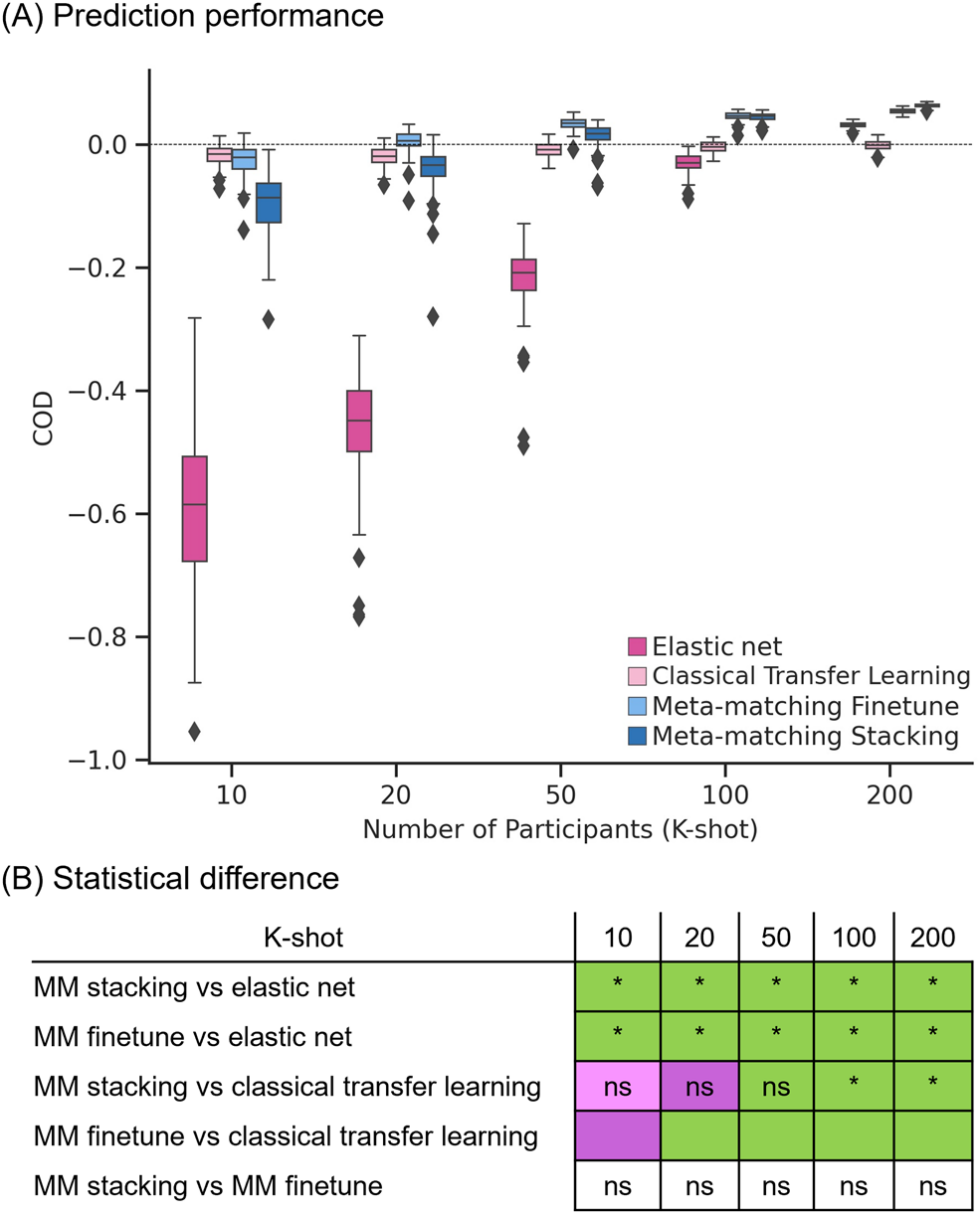
Meta-matching compared favorably with elastic net and direct transfer learning in terms of coefficient of determination (COD) within the UK Biobank. (A) Phenotypic prediction performance (COD) averaged across 34 meta-test phenotypes in the UK Biobank. X-axis represents the number of participants in the meta-test set of the UK Biobank used to train an elastic net or adapt the pretrained model from the meta-training set of the UK Biobank. Each boxplot shows the distribution of performance over 100 repetitions of sampling K participants. (B) Statistical difference between the prediction performance (COD) of baseline methods and meta-matching algorithms. p Values were calculated based on a two-sided bootstrapping test. “*” indicates statistical significance after multiple comparisons correction (FDR q < 0.05). “ns” indicates statistical test did not survive FDR correction. We note that there was no statistical test between meta-matching finetune and classical transfer learning because the bootstrapping procedure was too expensive for the two methods. Colors indicate effect sizes of differences (Cohen’s D) between approaches. Light green indicates effect size ≥ 0.8. Dark green indicates 0 ≤ effect size < 0.8. Dark pink indicates −0.8 < effect size < 0. Light pink indicates effect size ≤ −0.8. There is no color for the comparison between meta-matching finetune and stacking since they are both our proposed methods.

In the case of Pearson’s correlation (Fig. 5), both meta-matching finetune and meta-matching stacking greatly outperformed elastic net and classical transfer learning for all values of K. Meta-matching stacking was statistically better than meta-matching finetune for K ≥ 100.

In the case of COD (Fig. 6), both meta-matching finetune and meta-matching stacking greatly outperformed elastic net for all values of K. For K ≤ 20, classical transfer learning was numerically better but not statistically better than meta-matching stacking. From K ≥ 50, meta-matching stacking was numerically better than transfer learning with statistical significance from K = 100 onward.

On the other hand, for K = 10, classical transfer learning was numerically better than meta-matching finetune, while meta-matching finetune was better than classical transfer learning for the remaining other values of K with large effect sizes (light green inFig. 6B). We note that there was no statistical test between meta-matching finetune and classical transfer learning because of the huge computational cost of the two approaches, so no bootstrapping was performed for either approach.

Another relevant point is that COD for all approaches was negative for K = 10. COD was positive for meta-matching finetune for K = 20 onward, and positive for meta-matching stacking for K = 50 onward. This suggests that absolute prediction accuracy (i.e., COD) is difficult even with meta-learning or transfer learning, when the sample size is very small.

Overall, meta-matching was better than elastic net for all values of K for both evaluation metrics (Pearson’s correlation and COD). On the other hand, meta-matching compared favorably with respect to transfer learning for all values of K for Pearson’s correlation and for larger values of K for COD.

### 3.2 Meta-matching outperforms baselines in the HCP-YA dataset

The previous experiment results (Figs. 5and6) suggest that meta-matching can perform well when transferring within the same dataset (UK Biobank). We now evaluate the generalizability of meta-matching across datasets, using the HCP-YA and HCP-Aging datasets (Fig. 2) in the following section and next section, respectively.

**Fig. 7.**
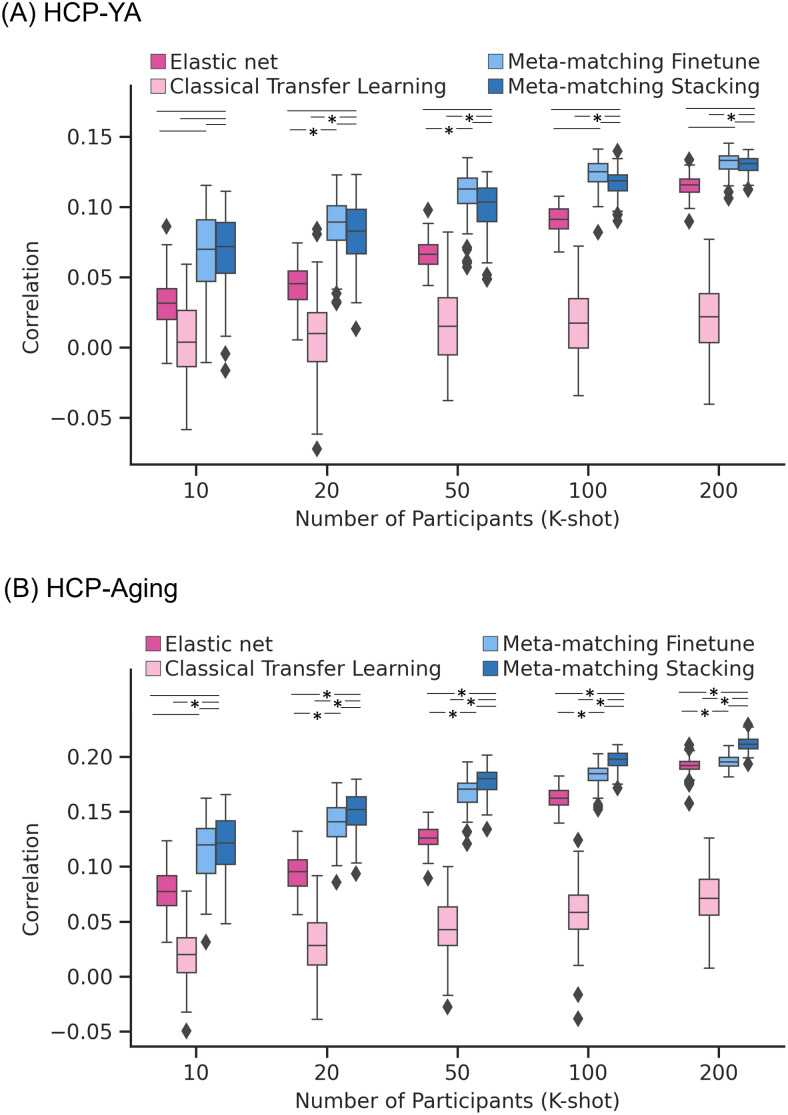
Meta-matching compared favorably with elastic net and classical transfer learning in terms of Pearson’s correlation when translating models from the UK Biobank to new target datasets. (A) Phenotypic prediction performance (Pearson’s correlation) averaged across 35 meta-test phenotypes in the HCP-YA dataset. X-axis represents the number of participants from the HCP-YA dataset used to train an elastic net or adapt the pretrained model from the meta-training set. Each boxplot shows the distribution of performance over 100 repetitions of sampling K participants. (B) Same plot as panel (A) except that the analyses were performed in the HCP-Aging dataset and averaged across the 45 meta-test HCP-Aging phenotypes. “*” and dash line indicate the results of a two-sided bootstrapping statistical test between meta-matching variants and other approaches. “*” indicates statistical significance after multiple comparisons correction (FDR q < 0.05). The dash line (without the “*”) indicates that the comparison was not significant. We note that there was no statistical test between meta-matching finetune and classical transfer learning because the bootstrapping procedure was too expensive for the two methods.

Figures 7Aand8Ashow the Pearson’s correlation and COD, respectively, averaged across all 35 meta-test phenotypes in the HCP-YA dataset. Each boxplot represents 100 random samplings of K participants.Figure 9Ashows the outcomes of the statistical tests obtained by a bootstrapping procedure (Supplementary Methods S2 and Figure S2). The actual p values are reported inTable S7. Colors indicate effect sizes of differences (Cohen’s D) between approaches.

**Fig. 8.**
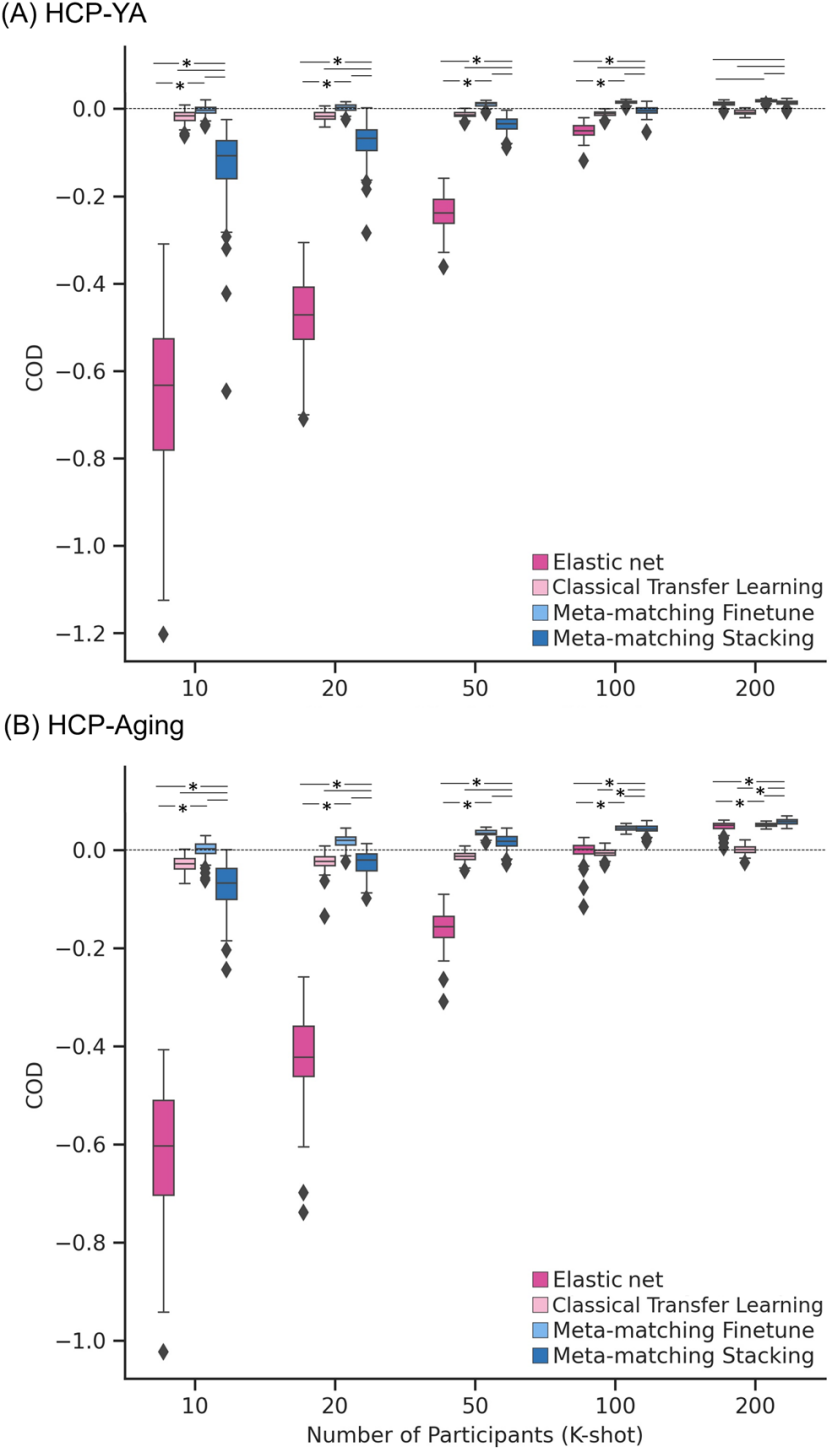
Meta-matching compared favorably with elastic net and classical transfer learning in terms of coefficient of determination (COD) when translating models from the UK Biobank to new target datasets. (A) Phenotypic prediction performance (COD) averaged across 35 meta-test phenotypes in the HCP-YA dataset. X-axis represents the number of participants from the HCP-YA dataset used to train an elastic net or adapt the pretrained model from the meta-training set. Each boxplot shows the distribution of performance over 100 repetitions of sampling K participants. (B) Same plot as panel (A) except that the analyses were performed in the HCP-Aging dataset and averaged across the 45 meta-test HCP-Aging phenotypes. “*” and dash line indicate the results of a two-sided bootstrapping statistical test between meta-matching variants and other approaches. “*” indicates statistical significance after multiple comparisons correction (FDR q < 0.05). The dash line (without the “*”) indicates that the comparison was not significant. We note that there was no statistical test between meta-matching finetune and classical transfer learning because the bootstrapping procedure was too expensive for the two methods.

**Fig. 9.**
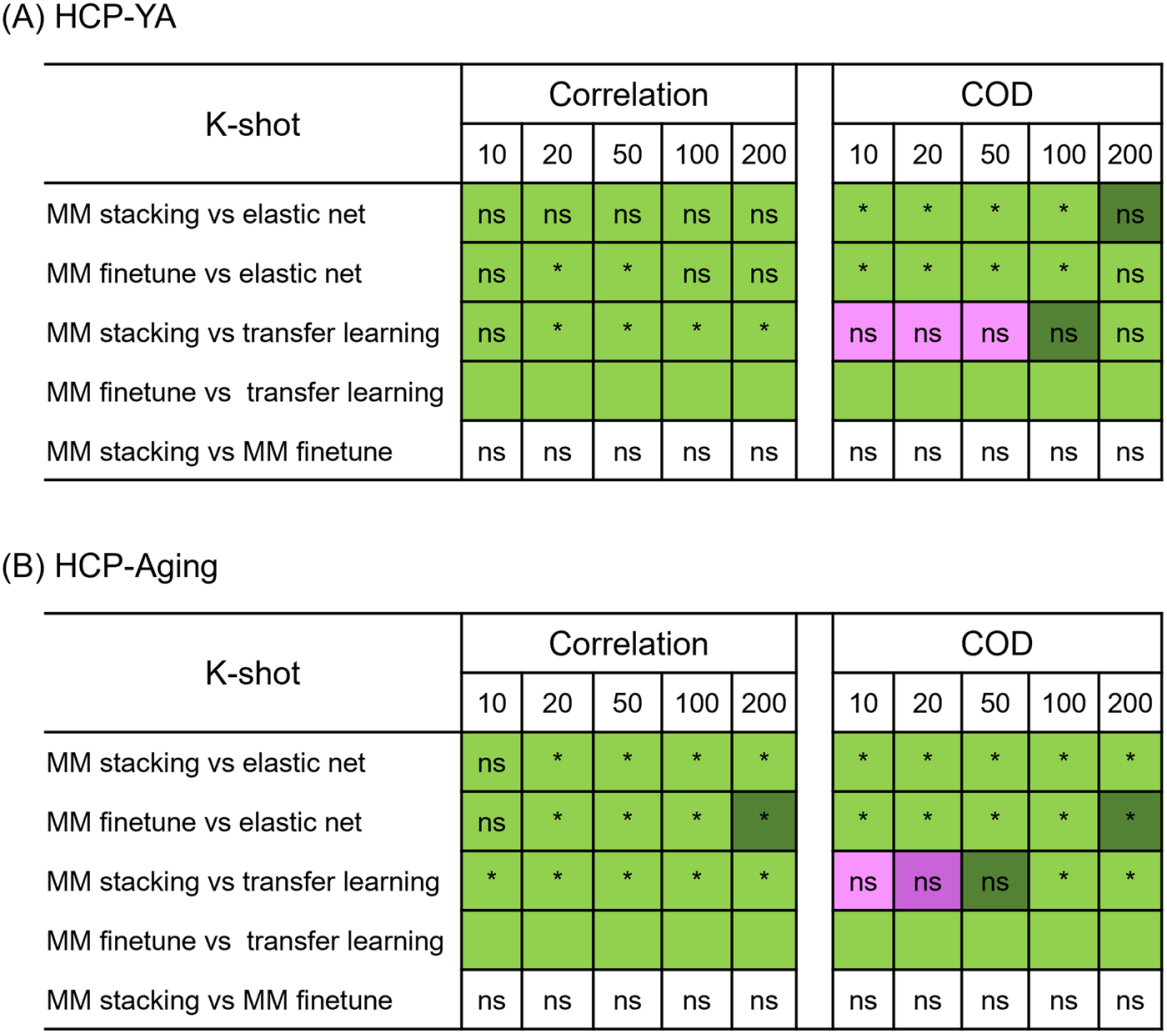
Statistical difference and effect size between the prediction performance of baseline methods and meta-matching algorithms for the (A) HCP-YA and (B) HCP-Aging datasets. p Values were calculated based on a two-sided bootstrapping test. “*” indicates statistical significance after multiple comparisons correction (FDR q < 0.05). “ns” indicates that statistical test did not survive FDR correction. We note that there was no statistical test between meta-matching finetune and classical transfer learning because the bootstrapping procedure was too expensive for the two methods. Colors indicate effect sizes of differences (Cohen’s D) between approaches. Light green indicates effect size ≥ 0.8. Dark green indicates 0 ≤ effect size < 0.8. Dark pink indicates −0.8 < effect size < 0. Light pink indicates effect size ≤ −0.8. There is no color for the comparison between meta-matching finetune and stacking since they are both our proposed methods.

In the case of Pearson’s correlation (Fig. 7A), both meta-matching finetune and meta-matching stacking were better than elastic net and classical transfer learning for all values of K with large effect sizes (light green inFig. 9A). Meta-matching finetune was statistically better than elastic net for K = 20 and 50. Meta-matching stacking was statistically better than classical transfer learning for K ≥ 20. For this cross-dataset analysis, meta-matching finetune was generally numerically better, but not statistically better than meta-matching stacking.

In the case of COD (Fig. 8A), both meta-matching finetune and meta-matching stacking greatly outperformed elastic net for K ≤ 100. For K ≤ 50, classical transfer learning was numerically better than meta-matching stacking with large effect sizes (light pink inFig. 9A), but the differences were not significant. From K ≥ 100, meta-matching stacking was numerically better, but not statistically better than transfer learning.

On the other hand, for all values of K, meta-matching finetune was numerically better than classical transfer learning with large effect sizes (light green inFig. 9A). We note that there was no statistical test between meta-matching finetune and classical transfer learning because of the huge computational cost of the two approaches, so no bootstrapping was performed for either approach.

Another relevant point is that COD for all approaches was negative (or almost zero) for K = 10, and only positive for meta-matching finetune for K ≥ 20, suggesting that absolute prediction accuracy (i.e., COD) is difficult even with meta-learning or transfer learning when the sample size is very small.

Overall, meta-matching compared favorably with respect to elastic net for all values of K for both evaluation metrics (Pearson’s correlation and COD). On the other hand, meta-matching compared favorably with respect to transfer learning for all values of K for Pearson’s correlation and for K ≥ 100 for COD.

### 3.3 Meta-matching outperforms baselines in the HCP-Aging dataset

Figures 7Band8Bshow the Pearson’s correlation and COD, respectively, averaged across all 45 meta-test phenotypes in the HCP-Aging dataset. Each boxplot represents 100 random samplings of K participants.Figure 9Bshows the outcomes of the statistical tests obtained by a bootstrapping procedure (Supplementary Methods S2 and Figure S3). The actual p values are reported inTable S8. Colors indicate effect sizes of differences (Cohen’s D) between approaches.

In the case of Pearson’s correlation (Fig. 7B), both meta-matching finetune and meta-matching stacking greatly outperformed elastic net and classical transfer learning for most values of K. Meta-matching stacking was statistically better than elastic net for K ≥ 20. Meta-matching stacking was statistically better than classical transfer learning for all values of K. For this cross-dataset analysis, meta-matching stacking was numerically better, but not statistically better than meta-matching finetune.

In the case of COD (Fig. 8B), both meta-matching finetune and meta-matching stacking greatly outperformed elastic net for all values of K. For K ≤ 20, classical transfer learning was numerically better, but not statistically better than meta-matching stacking. From K ≥ 50, meta-matching stacking was numerically better than transfer learning with statistical significance achieved for K ≥ 100.

On the other hand, for all values of K, meta-matching finetune was numerically better than classical transfer learning with large effect sizes (light green inFig. 9B). We note that there was no statistical test between meta-matching finetune and classical transfer learning because of the huge computational cost of the two approaches, so no bootstrapping was performed for either approach.

Another relevant point is that COD for all approaches was negative (or almost zero) for K = 10, and only positive for meta-matching finetune for K ≥ 20, suggesting that absolute prediction accuracy (i.e., COD) is difficult even with meta-learning or transfer learning when the sample size is very small.

Overall, meta-matching was better than elastic net for all values of K for both evaluation metrics (Pearson’s correlation and COD). On the other hand, meta-matching compared favorably with respect to transfer learning for all values of K for Pearson’s correlation and for K ≥ 50 for COD.

### 3.4 Different improvements on different phenotypes

**Fig. 10.**
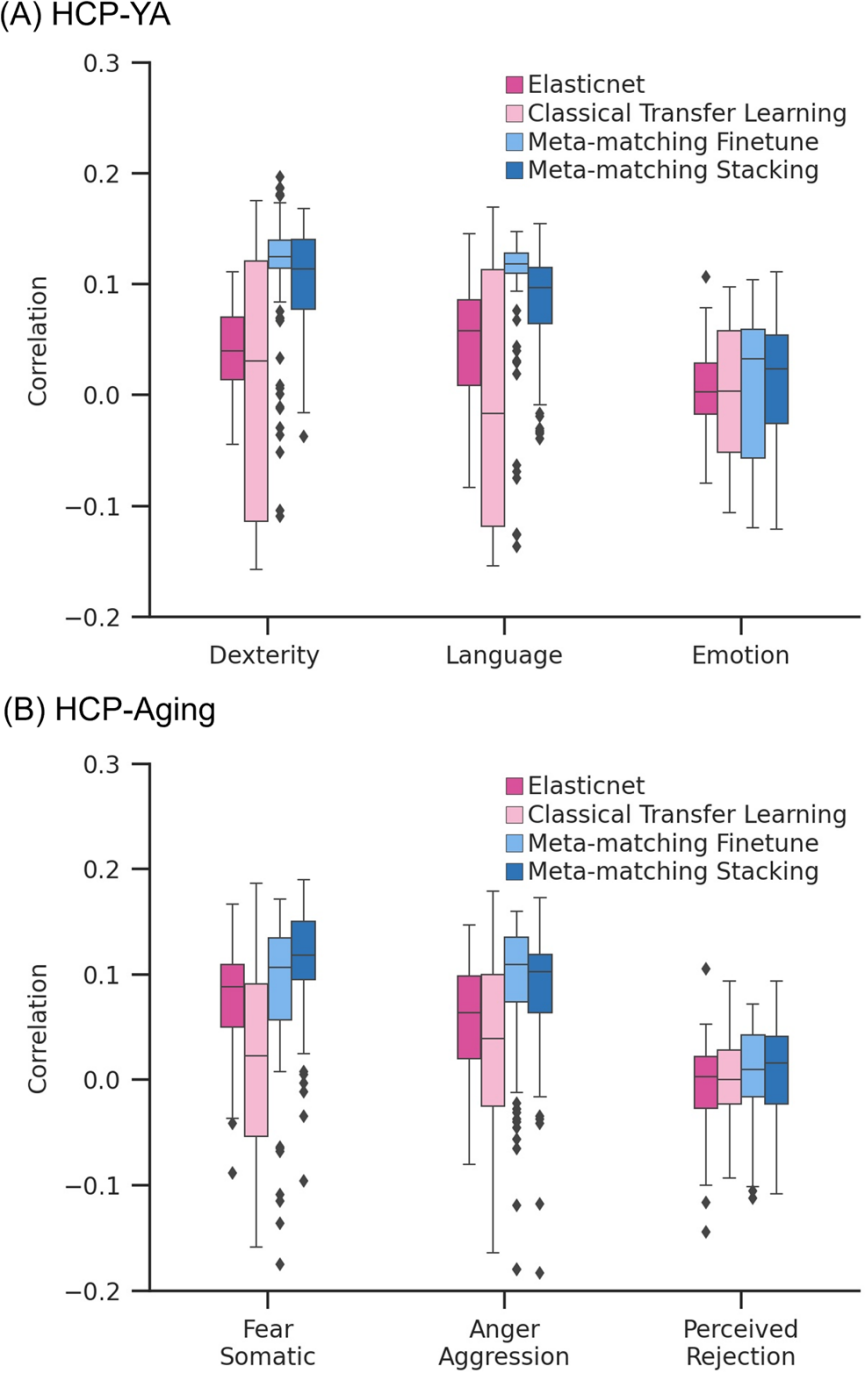
Examples of prediction performance (Pearson’s correlation) for different nonbrain-imaging phenotypes in the (A) HCP-YA and (B) HCP-Aging datasets in the case of K = 100 participants.

Overall, meta-matching improved prediction on average across multiple phenotypes. However, we note that the improvement was not uniform across phenotypes.Figure 10illustrates the prediction performance (Pearson’s correlation) of three nonbrain-imaging phenotypes for K = 100 participants. In the case of the HCP-YA dataset (Fig. 10A), meta-matching finetune compared favorably with other approaches for predicting dexterity and language, but only achieved similar prediction accuracy on emotion. In the case of the HCP-Aging dataset (Fig. 10B), meta-matching stacking compared favorably with other approaches for predicting fear somatic and anger aggression, but only achieved similar prediction accuracy on perceived rejection.Tables S9 and S10report the prediction accuracy (Pearson’s correlation and COD) of all phenotypes in the HCP-YA and HCP-Aging datasets for all approaches.Tables S11 and S12report the prediction errors (mean absolute error) for all phenotypes.

**Fig. 11.**
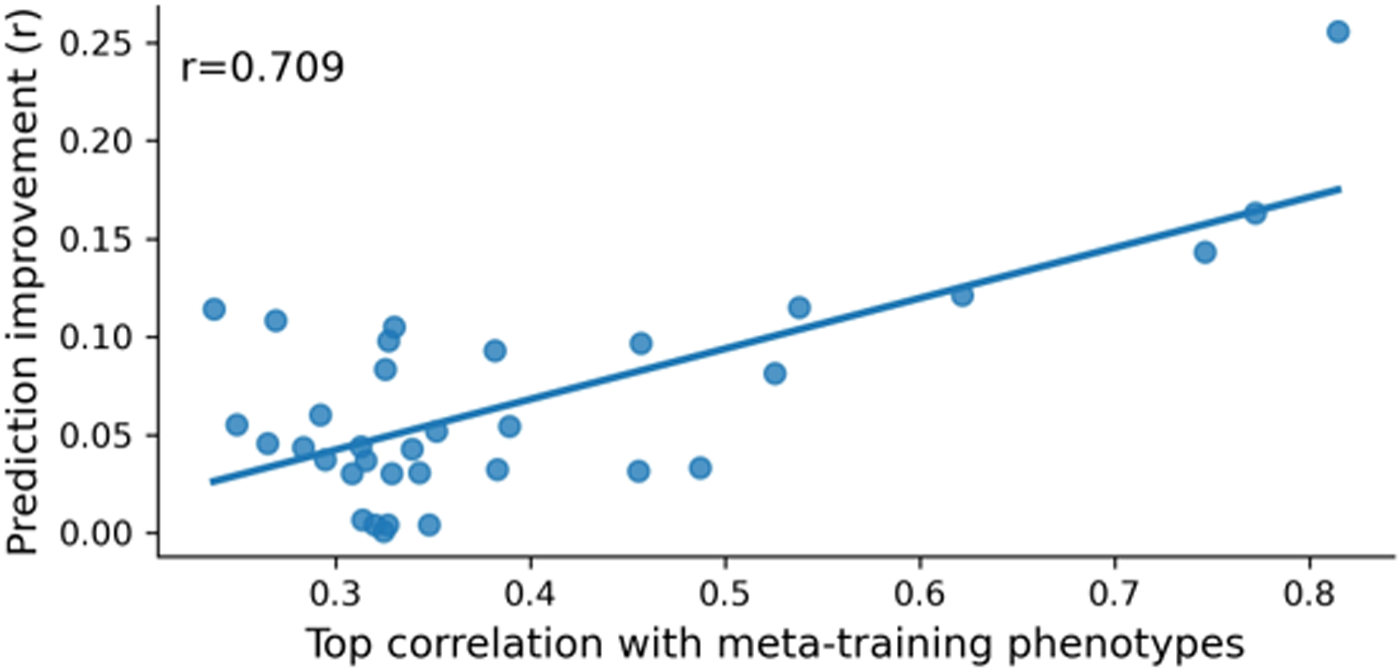
Prediction improvements were driven by correlations between meta-training and meta-test phenotypes. Vertical axis shows the prediction improvement of meta-matching stacking with respect to elastic net baseline under the 100-shot scenario. Prediction performance was measured using Pearson’s correlation. Each dot represents a meta-test phenotype. Horizontal axis shows each test phenotype’s top absolute Pearson’s correlation with phenotypes in the meta-training set. Test phenotypes with stronger correlations with at least one training phenotype led to greater prediction improvement with meta-matching.

Given that meta-matching exploits correlations among phenotypes, we hypothesized that variability in prediction improvements was driven by interphenotype correlations between the meta-training and meta-test sets.Figure 11shows the performance improvement (Pearson’s correlation) of meta-matching stacking as a function of the maximum correlation between each meta-test phenotype and meta-training phenotype in the within-UK Biobank analysis. As expected, meta-test phenotypes with stronger correlations with at least one meta-training phenotype led to greater prediction improvement with meta-matching. We note that this analysis required meta-training and meta-test phenotypes to be present in the same participants, so could only be performed for the within-UK Biobank analysis.

### 3.5 Interpreting meta-matching stacking with Haufe transform

**Fig. 12.**
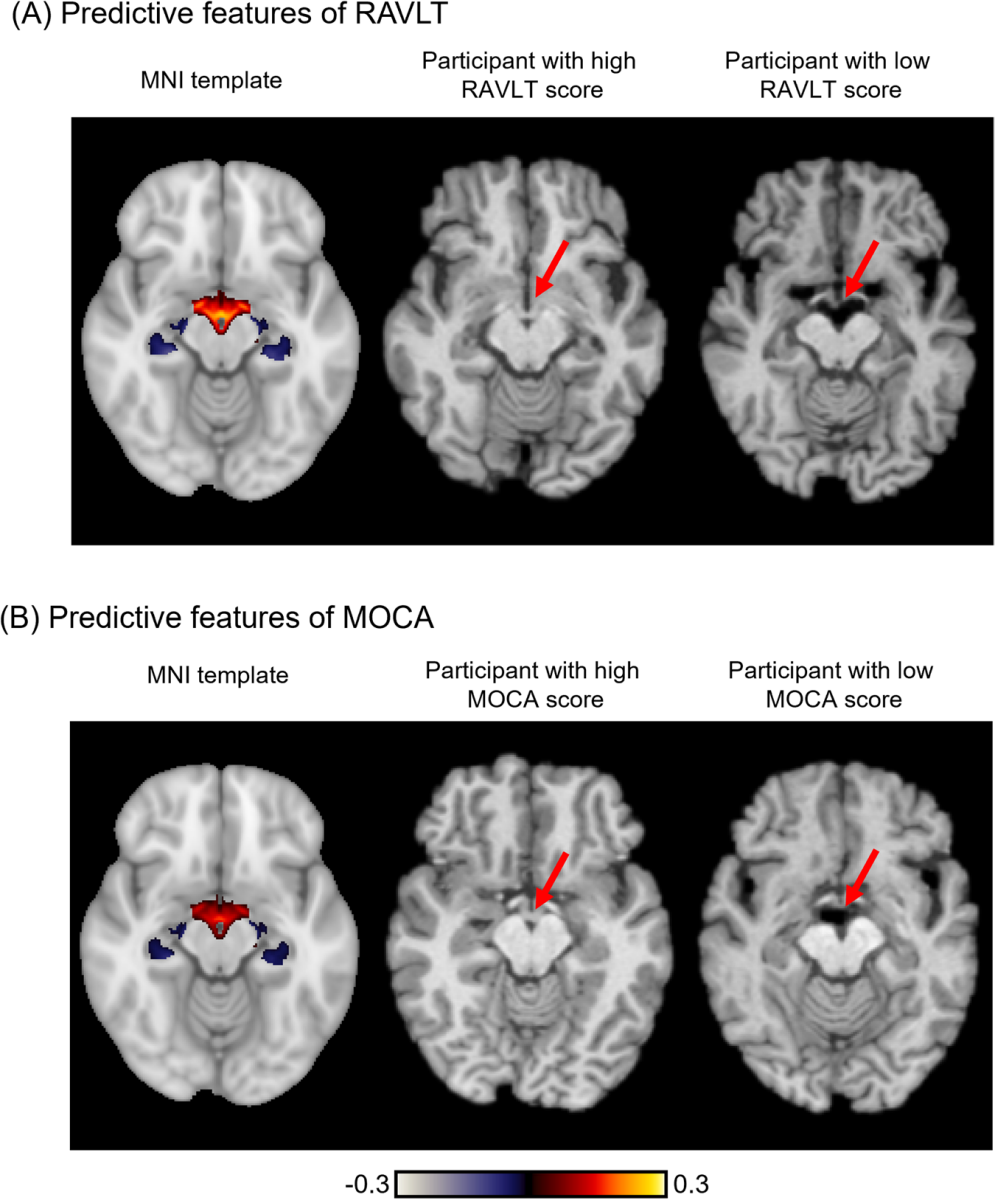
Feature importance of meta-matching stacking in the HCP-Aging dataset for K = 100 participants. (A) Feature importance map of meta-matching stacking from predicting Rey Auditory Verbal Learning Test (RAVLT) score. Left panel shows the feature importance map on the MNI152 template. A positive (or negative) feature importance value indicates that higher intensity was associated with predicting greater (or lower) phenotypic values. Middle panel shows an example participant with high RAVLT score. Right panel shows an example participant with low RAVLT score. (B) Feature importance map of meta-matching stacking from predicting the Montreal Cognitive Assessment (MOCA) score. Left panel shows the feature importance map on the MNI152 template. Middle panel shows an example participant with high MOCA score. Right panel shows an example participant with low MOCA score.

Figure 12illustrates the feature importance maps obtained from the Haufe transform for predicting the Rey Auditory Verbal Learning Test (RAVLT) score and Montreal Cognitive Assessment (MOCA) in the HCP-Aging dataset for K = 100. We note that a higher RAVLT or MOCA scores indicated better cognition.

Since we are using T1 intensity for prediction, linking the feature importance values of T1 intensities to more neuroanatomically grounded interpretations has to be done with care. For both RAVLT and MOCA, positive feature importance values were observed in the ventral diencephalon and the third ventricle (left panels ofFigure 12Aand12B), which suggested that higher T1 value led to prediction of better cognition (higher RAVLT and MOCA scores). By observing participants who performed poorly (right panels ofFigure 12Aand12B) and participants who performed well (middle panels ofFigure 12Aand12B), we inferred that the prediction might be partially driven by enlarged ventricles in participants with worse cognition (arrows inFig. 12), yielding a lower T1 value in the region.

Similarly, we observed negative feature importance values on the edges of the left and right hippocampi (rather than directly on top of the hippocampi), which suggested that higher T1 value led to prediction of worse cognition (lower RAVLT and MOCA scores). By observing participants who performed poorly (right panels ofFigure 12Aand12B) and participants who performed well (middle panels ofFig. 12Aand12B), we inferred that the prediction might be partially driven by gray-matter loss at or near the hippocampi, yielding a higher T1 value in the region, consistent with the aging literature (Apostolova et al., 2012;Ritter et al., 2017).

## 4 Discussion

In this study, we adapted two meta-matching variants from our previous study (He et al., 2022) to translate prediction models trained from large-scale T1-weighted anatomical MRI datasets to predict new nonbrain-imaging phenotypes in small-scale T1-weighted anatomical MRI datasets. We demonstrated that meta-matching finetune and meta-matching stacking greatly outperformed classical elastic net and classical transfer learning when the number of participants ≤ 200. Meta-matching performed well even when translating from a large-scale dataset (UK Biobank) to a small dataset (HCP-YA or HCP-Aging) with different scanners, acquisition, demographics, and preprocessing.

### 4.1 Benchmarking

Across all analyses in the UK Biobank, HCP-YA, and HCP-Aging datasets (Figs. 5 to 9), meta-matching consistently outperformed elastic net across both evaluation metrics (correlation and COD). It is worth noting that the elastic net utilized thickness and volumetric measures generated by FreeSurfer, instead of the intensity values of T1 images (like meta-matching and transfer learning). One reason is that it is too computationally expensive to include a baseline elastic net with T1 image as input. Based on our back of the envelope calculations, just running such a baseline on HCP-Aging alone would require around 50 days of computation time on our computing facility. Furthermore, given that we are working in the small sample regime with K ≤ 200 training participants, we believe that the small number of less than 200 predefined morphometric features together with elastic net provides a stronger baseline than an elastic net with T1 image as the input.

When using Pearson’s correlation as an evaluation metric, transfer learning performed poorly with substantially worse performance than both meta-matching variants and even elastic net (Figs. 5,7and9). On the other hand, when using COD as an evaluation metric, transfer learning was more competitive with respect to the other approaches (Figs. 6,8, and9). More specifically, transfer learning was numerically better (but not statistically better) than meta-matching stacking for small values of K, while meta-matching stacking was numerically better (and sometimes statistically better) than transfer learning for larger values of K.

On the other hand, meta-matching finetune outperformed transfer learning for most values of K even in the case of COD. We note that meta-matching finetune is similar to classical transfer learning in the sense that the last two layers of the CNN were finetuned. However, while transfer learning initialized the last layer of the CNN from scratch (Section 2.3.2), meta-matching finetune retained the weights leading to the output node that predicted the K meta-test participants the best (for each meta-test phenotype). This further supported the importance of the meta-matching approach.

Overall, meta-matching stacking was the best for the Pearson’s correlation metric, while meta-matching finetune was the best for COD. Pearson’s correlation is a measure of relative prediction performance, while COD is a measure of absolute prediction performance (E. S. Finn et al., 2015;Poldrack et al., 2020;Scheinost et al., 2019). Therefore, researchers more focused on relative prediction performance might consider using meta-matching stacking, while researchers more focused on absolute prediction performance might consider using meta-matching finetune. Furthermore, all approaches achieved negative or close to zero COD when K ≤ 20, suggesting that absolute prediction remains out of reach in the very small sample regime. COD was above zero for meta-matching finetune when K ≥ 50. However, COD values were still less than 0.1 (i.e., 10% of explained variance) even when K = 200, suggesting potential room for improvement.

However, we note that the reported averaged COD values obscured large variation in prediction accuracies across phenotypes. In the case of HCP-Aging, when K = 100 (Table S10), meta-matching finetune was able to achieve COD of more than 0.2 (i.e., more than 20% explained variance) for certain cognitive (e.g., processing speed) and physical (e.g., endurance) measures, while other phenotypes (e.g., positive affect) still could not achieve better than chance prediction (COD < 0).

We also observe that prediction accuracy was generally higher in the HCP-Aging dataset than in the HCP-YA dataset. One reason might be because the UK Biobank comprised middle-aged and elderly participants, so might generalize better to elderly participants in the HCP-Aging dataset than young adults in the HCP-YA dataset. A second reason might be that the relationship between interindividual variability in brain structure and phenotypic measures is stronger in elderly participants than in younger adults because of the well-known effects of aging on brain structure (Jockwitz et al., 2019;Kuznetsova et al., 2016). Clear evidence for the second reason comes from the fact that the elastic net baseline also performed better in the HCP-Aging dataset than in the HCP-YA dataset.

### 4.2 Interpreting meta-matching models

Meta-matching models can be interpreted at the level of imaging features by using the Haufe transform (Haufe et al., 2014). To illustrate this procedure, we applied the Haufe transform (Haufe et al., 2014) to the translated meta-matching stacking models in the HCP-Aging dataset (Fig. 12). For a given meta-test phenotype, Haufe transform was calculated as the covariance between the phenotype’s prediction based on the meta-matching stacking model and the intensity value of each T1 voxel (across K participants), yielding a 3D volume. We found that poorer cognitive performance in terms of worse RAVLT and MOCA scores was related to greater gray-matter atrophy and larger ventricular size, which is consistent with the aging literature (Apostolova et al., 2012;Ritter et al., 2017). Meta-matching finetune can be interpreted in a similar fashion.

In addition to interpreting meta-matching models at the level of brain-imaging features, the meta-matching models can also be interpreted at the level of phenotypic traits. In the case of meta-matching stacking, this can again be achieved using the Haufe transform. To illustrate this, let us consider the pretrained 3D CNN model from the UK Biobank with 67 prediction outputs. This 3D CNN model can be translated to predict a new meta-test phenotype using K participants from the meta-test set using the stacking procedure. The Haufe transform can then be calculated as the covariance (across the K participants) between the phenotype’s prediction from the final stacking model and the 67 inputs to the stacking model, yielding a vector of length 67, which indicates the relative importance of the original 67 meta-training phenotypes for predicting the meta-test phenotype.

### 4.3 Limitations and future work

Because meta-matching exploits correlations between the phenotypes of meta-training and meta-test sets, the amount of prediction improvement strongly relied on the strongest correlations between the meta-test phenotype and meta-training phenotypes (Fig. 11). Consequently, not all phenotypes might benefit from meta-matching. However, we note that this limitation exists for all meta-learning and transfer learning algorithms—model transfer is easier if the source and target domains are more similar; performance will degrade if source and target domains are very different.

The mismatch between meta-test and meta-training phenotypes can be accentuated by population differences or lack of diversity in the meta-training set (Greene et al., 2022;Li et al., 2022). Since our current study utilized a single source dataset (UK Biobank), the meta-matching models might not generalize as well to new populations, e.g., of different ethnicity or age. In our study, meta-matching still outperformed classical elastic net in young adults from North America (HCP-YA datasets), but we could potentially achieve even better results if meta-matching models were trained on a wider range of source datasets that included young adults from North America. An important future direction is to develop meta-matching models based on multiple diverse T1 datasets. We have recently developed meta-matching variants (dataset stacking and multilayer meta-matching) for resting-state functional connectivity (Chen et al., 2023), which could be adapted to T1 data.

Beyond RSFC from our previous studies (Chen et al., 2023;He et al., 2020) and T1 measures considered in this study, we can in principle extend meta-matching to other MRI modalities (e.g., diffusion MRI) and non-MRI data (e.g., electroencephalogram). Other T1 measures, such as surface area, sulcal depth, and curvature, could also be considered. Fusion of meta-matching models across modalities might potentially improve prediction performance, although we note that such an improvement is not a guaranteed outcome. For example, our previous study (Ooi et al., 2022) found that integrating T1, diffusion, and functional connectivity measures within a multikernel regression framework did not improve prediction accuracy in young healthy participants over just functional connectivity alone. However, this negative finding (Ooi et al., 2022) might not generalize to meta-matching and other populations. One tricky issue arising from multiple modality fusion is dealing with inevitable missing modality in a new test participant, which is still an unsolved problem.

Finally, in the current study, we trained our meta-matching model based on the FSL MNI152 template space because it is the most popular standard space in the literature and also because the major datasets (in this case, UK Biobank, HCP-Aging, HCP-YA) provide T1 data in that space. Researchers using other volumetric coordinate systems, e.g., Colin27 (Holmes et al., 1998) and SPM MNI152 (Mazziotta et al., 2001), might not be able to benefit as much from our models. However, unlike different RSFC atlases (Craddock et al., 2012;Gordon et al., 2016;Schaefer et al., 2018;Shen et al., 2013;Yan et al., 2023;Yeo et al., 2011), the different volumetric spaces are probably not as different. As such, it is entirely possible that our meta-matching procedure on the K participants in other coordinate systems (e.g., Colin27 or SPM MNI152) might overcome atlas space differences, yielding good performance. We leave this to future work.

### 4.4 Related studies

There has been a large number of studies using T1 MRI to predict individual-level phenotypic traits, clinical symptoms, and diagnostic categories (Arbabshirani et al., 2017;Bhagwat et al., 2019;Cohen et al., 2021;Ooi et al., 2022;Sabuncu et al., 2015). However, most of these studies focused on within-dataset prediction, without considering the generalization of their predictive models to new datasets (Wu et al., 2023). In more recent years, there is a growing number of studies adapting models trained on large datasets to predict the same phenotype in new data (Holderrieth et al., 2022;Jónsson et al., 2019), and more rarely, to predict a new phenotype in a new dataset (Leonardsen et al., 2022;Lu et al., 2022). This type of transfer learning or meta-learning is typically achieved by some form of finetuning of the model trained on one or more large-scale source datasets (Bae et al., 2021;Dhinagar et al., 2023;Wood et al., 2024), similar to our transfer learning baseline. As shown in our study, both meta-matching variants appeared to outperform this type of transfer learning.

## 5 Conclusion

In this study, we showed that meta-matching can be used to translate T1-based phenotypic prediction models from large source datasets to predict new phenotypes in small target datasets. By exploiting correlations between phenotypes, meta-matching greatly outperformed elastic net and classical transfer learning, both when translating models within the same dataset and when translating models across datasets with different MRI scanners, acquisition protocols, and demographics. Overall, our results demonstrated the versatility of the meta-matching framework.

## Data and Code Availability

The code used in this study can be found here (https://github.com/ThomasYeoLab/CBIG/tree/master/stable_projects/predict_phenotypes/Naren2024_MMT1). Two coauthors (Lijun An and Chen Zhang) reviewed the code before merging it into the GitHub repository to reduce the chance of coding errors. The trained models for meta-matching are also publicly available (https://github.com/ThomasYeoLab/Meta_matching_models/tree/main/T1/v1.0). This study used publicly available data from the UK Biobank (https://www.ukbiobank.ac.uk/), as well as the HCP-YA and HCP-Aging datasets (https://www.humanconnectome.org/). Data can be accessed via data use agreements.

## Author Contributions

N.W., L.A., C.Z., R.K., P.C., D.B., S.B.E., A.J.H., and B.T.T.Y. designed the research. N.W. conducted the research. N.W., L.A., C.Z., R.K., P.C., D.B., S.B.E., A.J.H., and B.T.T.Y. interpreted the results. N.W. and B.T.T.Y. wrote the manuscript and made the figures. N.W., L.A., and C.Z. reviewed and published the code. All authors contributed to project direction via discussion. All authors edited the manuscript.

## Declaration of Competing Interest

The authors declare no competing interests.

## Supplementary Material

Supplementary Material
