# Supplementary material for: Translating phenotypic prediction models from big to small anatomical MRI data using meta-matching
Source: Imaging Neurosci (Camb). 2024 Aug 1;2:imag-2-00251. doi: 10.1162/imag_a_00251 (PMC12272219; doi:10.1162/imag_a_00251)
Supplement: Supplementary Material [file imag_a_00251-supp.pdf]

Supplementary Materials

This supplementary material is divided into Supplementary Methods and Supplementary Results to complement the Methods and Results sections in the main text, respectively.

Supplemental Methods

S1. Details of pre-training 3D-CNN on the UK Biobank

Details about the 3D CNN model (pre-trained on the UK Biobank) are shown below. We note that hyperparameters were empirically determined based on the meta-training set from the within-UK Biobank analysis (Figure 1A). Both within-UK Biobank and cross-dataset analyses used the same set of hyperparameters (Table S1).

During training (as well as when performing meta-matching finetune and transfer learning), mean square error was used as the loss function. The output layer of the 3D CNN model had 33 nodes for within UK Biobank analyses and 67 nodes for cross-dataset analyses. The maximum number of training epochs was 100 for training in the meta-training set. The initial learning rate was 1e-5 and decreased by a factor 10 after every 50 epochs. The meta-training validation set was used to evaluate the validation loss for early stopping.

The 3D CNN model was implemented using PyTorch (Paszke et al., 2017). We note that in pytorch, the convolutional layer pads zeros around the edges of the image before performing the convolution operation.

Hyperparameter	Value
Number of 3D convolutional blocks	6
Number of channels	[32 64 128 256 256 64]
Dropout rate	0.1
Starting learning rate	1e-5
Weight decay rate	0.02
Batch size	8

Table S1. Hyperparameters for the 3D CNN model.

S2. Bootstrapping procedure

Following our previous study (He et al., 2022), we used a bootstrapping procedure to evaluate whether there is a statistical difference between algorithms. The bootstrap sampling was repeated 1,000 times. For each bootstrap sampling, we randomly chose K participants in the meta-test set with replacement, and the remaining participants in the meta-test set were used as test participants. We applied elastic net and meta-matching stacking separately on each of the 1,000 bootstrapped samples, resulting in 1,000 COD and Pearson's correlation from the bootstrapped samples for each method. 1,000 bootstrapping was not performed for classical transfer learning and meta-matching finetune because it would have required 40 days of computing time (on a single GPU).

We calculated the statistical significance for Pearson's correlation and COD separately. For ease of illustration, we focused on COD to explain the procedure for the statistical test. To compute the statistical difference between two approaches with 1,000 bootstrapped samples (i.e., elastic net and meta-matching stacking), we fitted a Gaussian distribution to the difference of 1,000 COD between the two algorithms. We then calculated the p-value as follows:

$$p = \begin{cases} 2 \times \mathrm{CDF}(0) & \text{if } \mu \geq 0 \\ 2 \times \left(1 - \mathrm{CDF}(0)\right) & \text{if } \mu < 0 \end{cases} \quad (1)$$

CDF and μ are the cumulative distribution function and the mean of the Gaussian distribution separately.

To compute the statistical difference between one approaches with 1,000 bootstrapped samples and another approach with no bootstrapped samples (e.g., elastic net versus meta-matching finetune), we fitted a Gaussian distribution to the 1,000 COD of elastic net. We then calculated the p-value as follows:

$$p = \begin{cases} 2 \times \mathrm{CDF}(u_m) & \text{if } \mu \geq u_m \\ 2 \times \left(1 - \mathrm{CDF}(u_m)\right) & \text{if } \mu < u_m \end{cases} \quad (2)$$

CDF and μ are the cumulative distribution function and the mean of the Gaussian distribution separately. u_m is the average COD of meta-matching finetune across the 100 random repeats of K-shot. The procedure for Pearson's correlation is the same as COD. FDR (q < 0.05) is used for multiple comparisons correction.

S3. Coefficient of determination (COD)

The formula to calculate the COD in our study is shown below (Feilong et al., 2021):

$$\mathrm{COD} = 1 - \frac{\sum_{i=1}^{n}(y_i - \hat{y}_i)^2}{\sum_{i=1}^{n}(y_i - \bar{y})^2} \ ,$$

where y_i is the phenotypic value of the i-th test sample, $\hat{y}_i$ is the prediction of the phenotype for the i-th test sample, and $\bar{y}$ is the mean of phenotypic values across the K training participants.

UKBB field	Description
#household	number of people in household
#Mem C1	numeric memory principal component 1
Age	age
Age edu	age completed full time education
Alcohol 1	average monthly spirits intake
Alcohol 2	average weekly champagne plus white wine intake
Alcohol 3	average weekly beer plus cider intake
Blood C2	blood assays principal component 2
Blood C3	blood assays principal component 3
Blood C4	blood assays principal component 4
Blood C5	blood assays principal component 5
Body C1	anthropometry principal component 1
Body C2	anthropometry principal component 2
Body C3	anthropometry principal component 3
Bone C1	bone-densitometry of heel principal component 1
Bone C3	bone-densitometry of heel principal component 3
BP eye C2	blood pressure & eye measures component 2
BP eye C3	blood pressure & eye measures principal component 3
BP eye C4	blood pressure & eye measures principal component 4
BP eye C5	blood pressure & eye measures principal component 5
BP eye C6	blood pressure & eye measures principal component 6
Breath C1	spirometry principal component 1
Cancer C1	cancer principal component 1
Carotid C1	carotid ultrasound principal component 1
Carotid C5	carotid ultrasound principal component 5
Deprive C1	multiple deprivation principal component 1
Digit 1	symbol digit substitution principal component 1
Digit-o C1	symbol digit substitution online principal component 1
Digit-o C6	symbol digit substitution online principal component 6
Dur C1	process durations principal component 1
Dur C2	process durations principal component 2
Dur C4	process durations principal component 4

ECG C1	ECG measures principal component 1
ECG C2	ECG measures principal component 2
ECG C3	ecg measures principal component 3
ECG C6	ECG measures principal component 6
Family C1	family history (parent's age) principal component 1
Fluid Int.	fluid intelligence
Genetic C1	genetic principal components and heterozygosity principal component 1
Grip C1	hand grip strength principal component 1
Hearing	hearing signal-to-noise-ratio (snr) of triplet (left)
Illness C1	non-cancer illness principal component 1
Illness C4	non-cancer illness principal component 4
Loc C1	location principal component 1
Match	pairs matching
Match-o	pairs matching online
Matrix C1	matrix pattern completion principal component 1
Matrix C2	matrix pattern completion principal component 2
Matrix C3	matrix pattern completion principal component 3
Neuro	neuroticism score
ProMem C1	prospective memory principal component 1
RT C1	reaction time principal component 1
Sex	sex
Sex G C1	genotype sex inference principal component 1
Sex G C2	genotype sex inference principal component 2
Sleep	sleep duration per day
Smoke C1	smoke principal component 1
Time drive	time spent driving per day
Time TV	time spent watching television (tv) per day
Time walk	number of days walked 10+ minutes per week
Tower C1	tower rearranging principal component 1
Trail C1	trail making principal component 1
Trail-o C1	trail making online principal component 1
Trail-o C4	trail making online principal component 4
Travel	frequency of travelling from home to job workplace per week
Urine C1	urine assays principal component 1

Work	weekly length of working hour for main job

Table S1. Dictionary of 67 phenotypes in UK Biobank.

HCP field	Description
PicSeq_Unadj	Visual Episodic Memory
CardSort_Unadj	Cognitive Flexibility (DCCS)
Flanker_Unadj	Inhibition (Flanker Task)
PMAT24_A_CR	Fluid Intelligence (PMAT)
ReadEng_Unadj	Vocabulary (Pronunciation)
PicVocab_Unadj	Vocabulary (Picture Matching)
ProcSpeed_Unadj	Processing Speed
DDic_AUC_40K	Delay Discounting
VSPLOT_TC	Spatial Orientation
SCPT_SPEC	Sustained Attention – Spec.
ListSort_Unadj	Working Memory (List Sorting)
MMSE_Score	Cognitive Status (MMSE)
PSQI_Score	Sleep Quality (PSQI)
Endurance_Unadj	Walking Endurance
GaitSpeed_Unadj	Walking Speed
Dexterity_Unadj	Manual Dexterity
Strength_Unadj	Grip Strength
Taste_Unadj	Taste Intensity
Emotion_Task_Face_Acc	Emotional Face Matching
Language_Task_Math_Avg_Difficulty_Level	Arithmetic
Language_Task_Story_Avg_Difficulty_Level	Story Comprehension
Relational_Task_Acc	Relational Processing
WM_Task_Acc	Working Memory (N-back)
NEOFAC_A	Agreeableness (NEO)
NEOFAC_O	Openness (NEO)
NEOFAC_C	Conscientiousness (NEO)
NEOFAC_E	Extraversion (NEO)
AngAggr_Unadj	Anger – Aggression
FearAffect_Unadj	Fear – Affect
Sadness_Unadj	Sadness
LifeSatisf_Unadj	Life Satisfaction

MeanPurp_Unadj	Meaning & Purpose
Loneliness_Unadj	Loneliness
PercStress_Unadj	Perceived Stress
SelfEff_Unadj	Self-Efficacy

Table S2. Dictionary of 35 phenotypes in the HCP-YA dataset.

HCP-Aging field	Description
LifeSatisf	Life Satisfaction
MeanPurp	Meaning & Purpose
PosAffect	Positive Affect
Sadness	Sadness
FearAffect	Fear – Affect
FearSomat	Fear – Somatic
AngAffect	Anger – Affect
AngHostil	Anger – Hostility
AngAggr	Anger – Aggression
ER40ANG	ER40 Correct Anger Identifications
ER40FEAR	ER40 Correct Fear Identifications
ER40NOE	ER40 Correct No Emotion Identifications
ER40SAD	ER40 Correct Sad Identifications
EmotSupp	Emotion Support
InstruSupp	Instrumental Support
Friendship	Friendship
Loneliness	Loneliness
PercReject	Perceived Rejection
PercHostil	Perceived Hostility
PercStress	Perceived Stress
SelfEff	Self-Efficacy
NEOFAC_A	Agreeableness (NEO)
NEOFAC_O	Openness (NEO)
NEOFAC_C	Conscientiousness (NEO)
NEOFAC_N	Neuroticism (NEO)
NEOFAC_E	Extraversion (NEO)
Endurance	Walking Endurance

GaitSpeed	Walking Speed
Strength	Grip Strength
PicSeq	Visual Episodic Memory
CardSort	Cognitive Flexibility (DCCS)
Flanker	Inhibition (Flanker Task)
ReadEng	Vocabulary (Pronunciation)
PicVocab	Vocabulary (Picture Matching)
ProcSpeed	Processing Speed
DDisc_AUC_40K	Delay Discounting
ListSort	Working Memory (List Sorting)
raw_vat	Raw Score for the Visual Acuity
raw_pain	Raw Score for the Pain Interference
winright_ncorr	Words in Noise Number Correct Right
psqi_total	Sleep Quality (PSQI)
tmta_raw	Trailmaking test part A: Time to completion
tmtb_raw	Trailmaking test Part B: Time to completion
pea_ravlt_sd_tc	RAVLT Short Delay Total Correct
moca_total	Montreal Cognitive Assessment Total Score

Table S3. Dictionary of 45 phenotypes on HCP-Aging dataset.

brain ROI thickness	brain ROI thickness
lh_caudalanteriorcingulate_thickness	rh_caudalanteriorcingulate_thickness
lh_caudalmiddlefrontal_thickness	rh_caudalmiddlefrontal_thickness
lh_cuneus_thickness	rh_cuneus_thickness
lh_entorhinal_thickness	rh_entorhinal_thickness
lh_fusiform_thickness	rh_fusiform_thickness
lh_inferiorparietal_thickness	rh_inferiorparietal_thickness
lh_inferiortemporal_thickness	rh_inferiortemporal_thickness
lh_insula_thickness	rh_insula_thickness
lh_isthmuscingulate_thickness	rh_isthmuscingulate_thickness
lh_lateraloccipital_thickness	rh_lateraloccipital_thickness
lh_lateralorbitofrontal_thickness	rh_lateralorbitofrontal_thickness
lh_lingual_thickness	rh_lingual_thickness
lh_medialorbitofrontal_thickness	rh_medialorbitofrontal_thickness

lh_middletemporal_thickness	rh_middletemporal_thickness
lh_paracentral_thickness	rh_paracentral_thickness
lh_parahippocampal_thickness	rh_parahippocampal_thickness
lh_parsopercularis_thickness	rh_parsopercularis_thickness
lh_parsorbitalis_thickness	rh_parsorbitalis_thickness
lh_parstriangularis_thickness	rh_parstriangularis_thickness
lh_pericalcarine_thickness	rh_pericalcarine_thickness
lh_postcentral_thickness	rh_postcentral_thickness
lh_posteriorcingulate_thickness	rh_posteriorcingulate_thickness
lh_precentral_thickness	rh_precentral_thickness
lh_precuneus_thickness	rh_precuneus_thickness
lh_rostralanteriorcingulate_thickness	rh_rostralanteriorcingulate_thickness
lh_rostralmiddlefrontal_thickness	rh_rostralmiddlefrontal_thickness
lh_superiorfrontal_thickness	rh_superiorfrontal_thickness
lh_superiorparietal_thickness	rh_superiorparietal_thickness
lh_superiortemporal_thickness	rh_superiortemporal_thickness
lh_supramarginal_thickness	rh_supramarginal_thickness
lh_transversetemporal_thickness	rh_transversetemporal_thickness

Table S4. Selected 62 brain ROI thicknesses for DKT40 atlas.

brain ROI volume	brain ROI volume
lh_caudalanteriorcingulate_volume	rh_postcentral_volume
lh_caudalmiddlefrontal_volume	rh_posteriorcingulate_volume
lh_cuneus_volume	rh_precentral_volume
lh_entorhinal_volume	rh_precuneus_volume
lh_fusiform_volume	rh_rostralanteriorcingulate_volume
lh_inferiorparietal_volume	rh_rostralmiddlefrontal_volume
lh_inferiortemporal_volume	rh_superiorfrontal_volume
lh_insula_volume	rh_superiorparietal_volume
lh_isthmuscingulate_volume	rh_superiortemporal_volume
lh_lateraloccipital_volume	rh_supramarginal_volume
lh_lateralorbitofrontal_volume	rh_transversetemporal_volume
lh_lingual_volume	3rd-Ventricle
lh_medialorbitofrontal_volume	4th-Ventricle
lh_middletemporal_volume	Left-Accumbens-area

lh_paracentral_volume	Right-Accumbens-area
lh_parahippocampal_volume	Left-Amygdala
lh_parsopercularis_volume	Right-Amygdala
lh_parsorbitalis_volume	Brain-Stem
lh_parstriangularis_volume	CC_Anterior
lh_pericalcarine_volume	CC_Central
lh_postcentral_volume	CC_Mid_Anterior
lh_posteriorcingulate_volume	CC_Mid_Posterior
lh_precentral_volume	CC_Posterior
lh_precuneus_volume	CSF
lh_rostralanteriorcingulate_volume	Left-Caudate
lh_rostralmiddlefrontal_volume	Right-Caudate
lh_superiorfrontal_volume	Left-Cerebellum-Cortex
lh_superiorparietal_volume	Right-Cerebellum-Cortex
lh_superiortemporal_volume	Left-Cerebellum-White-Matter
lh_supramarginal_volume	Right-Cerebellum-White-Matter
lh_transversetemporal_volume	EstimatedTotalIntraCranialVol
rh_caudalanteriorcingulate_volume	Left-Hippocampus
rh_caudalmiddlefrontal_volume	Right-Hippocampus
rh_cuneus_volume	Left-Inf-Lat-Vent
rh_entorhinal_volume	Right-Inf-Lat-Vent
rh_fusiform_volume	Left-Lateral-Ventricle
rh_inferiorparietal_volume	Right-Lateral-Ventricle
rh_inferiortemporal_volume	Optic-Chiasm
rh_insula_volume	Left-Pallidum
rh_isthmuscingulate_volume	Right-Pallidum
rh_lateraloccipital_volume	Left-Putamen
rh_lateralorbitofrontal_volume	Right-Putamen
rh_lingual_volume	Left-Thalamus-Proper
rh_medialorbitofrontal_volume	Right-Thalamus-Proper
rh_middletemporal_volume	Left-VentralDC
rh_paracentral_volume	Right-VentralDC
rh_parahippocampal_volume	WM-hypointensities
rh_parsopercularis_volume	Left-choroid-plexus
rh_parsorbitalis_volume	Right-choroid-plexus
rh_parstriangularis_volume	Left-vessel

rh_pericalcarine_volume	Right-vessel

Table S5. Selected 104 brain ROI volumes from DKT40 and ASEG atlases.

Supplemental Results

p-value		K=10	K=20	K=50	K=100	K=200
Correlation	MM stacking v.s. Elastic net	**7.17E-05**	**9.60E-08**	**2.78E-15**	**0**	**0**
	MM finetune v.s. Elastic net	**0.000109**	**3.07E-07**	**6.66E-16**	**0**	**1.55E-15**
	MM stacking v.s. Classical transfer learning	**1.62E-08**	**0**	**0**	**0**	**0**
	MM stacking v.s. MM finetune	0.3695	0.2112	0.1415	**0.0285**	**0.0003**
COD	MM stacking v.s. Elastic net	**0.0005**	**1.89E-06**	**1.78E-08**	**1.49E-06**	**6.65E-05**
	MM finetune v.s. Elastic net	**0.0001**	**4.68E-06**	**7.97E-08**	**9.02E-06**	**0.0031**
	MM stacking v.s. Classical transfer learning	0.0962	0.5311	0.1315	**4.85E-10**	**0**
	MM stacking v.s. MM finetune	0.1337	0.1680	0.1539	0.6837	0.0533

Table S6. Meta-matching outperformed elastic net and classical transfer learning in UK Biobank. Statistical differences among the different algorithms for both correlation and COD metrics. P values were calculated based on a bootstrapping procedure. 'MM' stands for 'meta-matching'. Bold denotes statistical difference after correction for multiple comparisons (FDR q < 0.05).

p-value		K=10	K=20	K=50	K=100	K=200
Correlation	MM stacking v.s. Elastic net	0.1561	0.0990	0.0650	0.1499	0.3829
	MM finetune v.s. Elastic net	0.0358	**0.0102**	**0.0051**	0.0337	0.2024
	MM stacking v.s. Classical transfer learning	0.0392	**0.0022**	**1.11E-07**	**0**	**0**
	MM stacking v.s. MM finetune	0.9631	0.7811	0.4498	0.2682	0.3337
COD	MM stacking v.s. Elastic net	**0.0151**	**0.0011**	**0.0010**	**0.0265**	0.2798
	MM finetune v.s. Elastic net	**0.0027**	**0.0001**	**5.70E-05**	**0.0012**	0.0557
	MM stacking v.s. Classical transfer learning	0.1799	0.1146	0.1874	0.9794	0.1431
	MM stacking v.s. MM finetune	0.1324	0.0384	**0.01226**	0.0300	0.1109

Table S7. Meta-matching outperformed elastic net and classical transfer learning in HCP-YA. Statistical differences among the different algorithms for both correlation and COD metrics. P values were calculated based on a bootstrapping procedure. ‘MM’ stands for ‘meta-matching’. Bold denotes statistical difference after correction for multiple comparisons (FDR q < 0.05).

p-value		K=10	K=20	K=50	K=100	K=200
Correlation	MM stacking v.s. Elastic net	0.0304	**0.0013**	**0.0001**	**0.0004**	**0.0043**
	MM finetune v.s. Elastic net	0.0451	**0.0035**	**0.0006**	**0.0025**	**0.0115**
	MM stacking v.s. Classical transfer learning	**9.16E-05**	**3.13E-09**	**0**	**0**	**0**
	MM stacking v.s. MM finetune	0.8949	0.7928	0.6398	0.4795	0.4527
COD	MM stacking v.s. Elastic net	**0.0047**	**2.84E-07**	**1.39E-06**	**0.0036**	**0.0116**
	MM finetune v.s. Elastic net	**0.0019**	**8.66E-08**	**7.20E-07**	**0.0023**	**0.0112**
	MM stacking v.s. Classical transfer learning	0.3717	0.7631	0.2004	**0.0009**	**1.25E-07**
	MM stacking v.s. MM finetune	0.1712	0.0997	0.1387	0.3173	0.5261

Table S8. Meta-matching outperformed elastic net and classical transfer learning in HCP-Aging. Statistical differences among the different algorithms for both correlation and COD metrics. P values were calculated based on a bootstrapping procedure. 'MM' stands for 'meta-matching'. Bold denotes statistical difference after correction for multiple comparisons (FDR $q < 0.05$).

	Pearson’s correlation				COD			
**HCP-YA phenotype**	**Elastic net**	**Transfer learning**	**MM finetune**	**MM stacking**	**Elastic net**	**Transfer learning**	**MM finetune**	**MM stacking**
**PicSeq_Unadj**	0.036	0.006	0.029	0.070	-0.02	-0.014	-0.012	-0.012
**CardSort_Unadj**	0.091	-0.019	0.129	0.114	-0.002	-0.018	0.011	-0.003
**Flanker_Unadj**	0.120	0.019	0.172	0.142	0.006	-0.007	0.025	0.005
**PMAT24_A_CR**	0.267	0.075	0.259	0.291	0.063	0.001	0.063	0.076
**ReadEng_Unadj**	0.215	0.055	0.258	0.271	0.039	-0.001	0.057	0.066
**PicVocab_Unadj**	0.231	0.019	0.254	0.255	0.046	-0.006	0.054	0.056
**ProcSpeed_Unadj**	0.058	-0.020	0.088	0.067	-0.015	-0.016	0.001	-0.015
**DDic_AUC_40K**	0.107	0.020	0.103	0.152	0.002	-0.008	0	0.009
**VSPLOT_TC**	0.272	0.016	0.274	0.271	0.066	-0.004	0.071	0.065
**SCPT_SPEC**	0.031	0.01	0.059	0.082	-0.023	-0.013	-0.011	-0.011
**ListSort_Unadj**	0.179	-0.005	0.176	0.153	0.022	-0.01	0.024	0.011
**MMSE_Score**	0.038	0	0.005	0.002	-0.016	-0.014	-0.01	-0.031
**PSQI_Score**	0.053	0.030	0.071	0.097	-0.011	-0.007	-0.007	-0.005
**Endurance_Unadj**	0.281	0.063	0.297	0.288	0.074	0.004	0.084	0.076
**GaitSpeed_Unadj**	0.042	-0.003	0.004	0.044	-0.014	-0.015	-0.015	-0.018
**Dexterity_Unadj**	0.058	0.028	0.126	0.126	-0.009	-0.007	0.002	0.003
**Strength_Unadj**	0.538	0.268	0.577	0.591	0.285	0.051	0.225	0.346
**Taste_Unadj**	0.125	0.058	0.169	0.143	0.002	-0.007	0.022	0.009
**Emotion_Task_Face_Acc**	0.012	-0.008	0.022	0.033	-0.026	-0.011	-0.016	-0.037
**Language_Task_Math_Avg_Difficulty_Level**	0.078	0.012	0.108	0.102	-0.005	-0.010	0.003	-0.002
**Language_Task_Story_Avg_Difficulty_Level**	0.218	0.047	0.247	0.235	0.039	-0.002	0.057	0.046
**Relational_Task_Acc**	0.145	0.028	0.177	0.200	0.013	-0.005	0.026	0.027
**WM_Task_Acc**	0.210	0.044	0.214	0.220	0.038	-0.001	0.043	0.037
**NEOFAC_A**	0.121	-0.032	0.141	0.128	0.003	-0.014	0.007	0.001
**NEOFAC_O**	0.096	0.01	0.113	0.119	-0.003	-0.01	0.004	0.001
**NEOFAC_C**	0.057	0	0.121	0.077	-0.011	-0.013	0.005	-0.009
**NEOFAC_E**	-0.002	0	0.037	0.028	-0.019	-0.011	-0.008	-0.026
**AngAggr_Unadj**	0.115	0.003	0.163	0.144	-0.001	-0.016	0.018	0.008
**FearAffect_Unadj**	0.011	-0.004	0.035	0.014	-0.020	-0.014	-0.011	-0.029
**Sadness_Unadj**	0.015	-0.001	-0.012	-0.025	-0.017	-0.012	-0.011	-0.036
**LifeSatisf_Unadj**	0.075	0.006	0.027	0.026	-0.013	-0.013	-0.012	-0.025
**MeanPurp_Unadj**	0.036	0.007	0.054	0.036	-0.015	-0.011	-0.007	-0.024
**Loneliness_Unadj**	0.003	-0.002	-0.013	-0.013	-0.025	-0.014	-0.019	-0.037
**PercStress_Unadj**	0.048	0.005	0.036	0.010	-0.015	-0.012	-0.013	-0.029
**SelfEff_Unadj**	0.065	-0.010	0.084	0.058	-0.01	-0.011	-0.005	-0.021

Table S9. Prediction performance (Pearson’s correlation & COD) for each non-brain-imaging phenotype in the HCP-YA dataset. Results are shown for K = 100 and are averaged across 100 random samplings of the 100 participants.

	Pearson’s correlation				COD			
**HCP-Aging phenotype**	**Elastic net**	**Transfer learning**	**MM finetune**	**MM stacking**	**Elastic net**	**Transfer learning**	**MM finetune**	**MM stacking**
**LifeSatisf**	0.166	0.059	0.235	0.240	0.018	-0.003	0.047	0.050
**MeanPurp**	0.008	-0.004	-0.017	-0.020	-0.014	-0.024	-0.007	-0.030
**PosAffect**	0.015	0	-0.007	0.031	-0.018	-0.024	-0.005	-0.016
**Sadness**	0.101	0.015	0.071	0.097	0.002	-0.016	-0.001	-0.005
**FearAffect**	0.124	0.041	0.111	0.137	0.01	-0.018	0.004	0.009
**FearSomat**	-0.027	-0.003	-0.010	0.016	-0.019	-0.023	-0.009	-0.018
**AngAffect**	0.130	0.016	0.132	0.167	0.008	-0.017	0.008	0.019
**AngHostil**	0.170	0.056	0.177	0.201	0.021	-0.007	0.022	0.029
**AngAggr**	0.095	0.017	0.124	0.107	0.002	-0.014	0.013	0
**ER40ANG**	0.230	0.105	0.272	0.280	0.046	0.005	0.061	0.07
**ER40FEAR**	0.104	0.029	0.075	0.152	0.001	-0.013	0.001	0.016
**ER40NOE**	0.068	-0.004	0.056	0.023	-0.008	-0.021	0	-0.020
**ER40SAD**	0.147	0.041	0.154	0.149	0.016	-0.014	0.014	0.012
**EmotSupp**	0.029	0.004	0.033	0.021	-0.015	-0.019	-0.003	-0.022
**InstruSupp**	0.077	0.026	0.079	0.081	-0.005	-0.015	0.002	-0.013
**Friendship**	0.068	0.008	0.065	0.058	-0.009	-0.017	-0.001	-0.015
**Loneliness**	0.124	0.022	0.117	0.135	0.010	-0.017	0.008	0.008
**PercReject**	0.008	-0.001	0.003	0.010	-0.017	-0.019	-0.007	-0.025
**PercHostil**	0.156	0.041	0.151	0.142	0.015	-0.014	0.014	0.006
**PercStress**	0.169	0.055	0.189	0.212	0.020	-0.008	0.027	0.035
**SelfEff**	0.025	-0.001	-0.003	0.041	-0.014	-0.020	-0.007	-0.019
**NEOFAC_A**	0.027	0.008	0.058	0.078	-0.014	-0.016	-0.001	-0.010
**NEOFAC_O**	0.099	0.008	0.098	0.163	0.003	-0.015	0.006	0.019
**NEOFAC_C**	-0.011	-0.001	-0.020	0.006	-0.017	-0.025	-0.007	-0.025
**NEOFAC_N**	0.134	0.047	0.145	0.144	0.011	-0.016	0.015	0.009
**NEOFAC_E**	-0.005	0.004	-0.016	0.012	-0.014	-0.018	-0.008	-0.017
**Endurance**	0.526	0.198	0.491	0.526	0.269	0.048	0.219	0.271
**GaitSpeed**	0.234	0.103	0.266	0.278	0.048	0.006	0.050	0.069
**Strength**	0.512	0.176	0.471	0.545	0.256	0.028	0.109	0.292
**PicSeq**	0.359	0.155	0.357	0.394	0.117	0.031	0.120	0.150
**CardSort**	0.341	0.097	0.351	0.347	0.109	0.004	0.109	0.113
**Flanker**	0.446	0.221	0.450	0.437	0.189	0.054	0.190	0.179
**ReadEng**	0.139	0.014	0.189	0.220	0.011	-0.017	0.024	0.040
**PicVocab**	0.229	0.079	0.257	0.307	0.042	-0.001	0.049	0.086
**ProcSpeed**	0.456	0.221	0.474	0.462	0.200	0.063	0.211	0.208
**DDisc_AUC_40K**	0.162	0.015	0.157	0.195	0.020	-0.016	0.019	0.030
**ListSort**	0.331	0.126	0.347	0.366	0.095	0.011	0.099	0.125
**raw_vat**	0.464	0.226	0.502	0.496	0.205	0.070	0.230	0.239
**raw_pain**	0.092	0.015	0.088	0.063	-0.002	-0.017	0.003	-0.014
**winright_ncorr**	0.488	0.272	0.530	0.540	0.228	0.101	0.257	0.284
**psqi_total**	0.113	-0.001	0.093	0.089	0.001	-0.020	0.005	-0.008
**tmta_raw**	0.441	0.198	0.477	0.483	0.176	0.062	0.208	0.221
**tmtb_raw**	0.380	0.148	0.384	0.394	-0.037	0.007	0.072	0.070
**pea_ravlt_sd_tc**	0.372	0.173	0.378	0.413	0.132	0.047	0.140	0.166
**moca_total**	0.300	0.115	0.273	0.297	0.082	0.011	0.055	0.080

Table S10. Prediction performance (Pearson’s correlation & COD) for each individual non-brain-imaging phenotype in the HCP-Aging dataset. Results are shown for K = 100 and are averaged across 100 random samplings of the 100 participants.

	MAE				Phenotypic values		
**HCP-YA phenotype**	**Elastic net**	**Transfer learning**	**MM finetune**	**MM stacking**	**Standard deviation**	**Minimum**	**Maximum**
**PicSeq_Unadj**	10.869	10.871	10.85	10.822	13.238	76.42	135.55
**CardSort_Unadj**	8.091	8.171	8.047	8.122	10.205	81.27	143.94
**Flanker_Unadj**	8.02	8.071	7.959	8.027	10.066	86.31	142.11
**PMAT24_A_CR**	3.761	3.942	3.791	3.725	4.689	4	24
**ReadEng_Unadj**	8.284	8.502	8.228	8.128	10.509	84.2	150.71
**PicVocab_Unadj**	7.347	7.545	7.293	7.282	9.557	92.04	153.089
**ProcSpeed_Unadj**	11.949	11.96	11.841	11.945	15.292	51.62	154.69
**DDic_AUC_40K**	0.246	0.249	0.247	0.244	0.285	0.016	0.984
**VSPLOT_TC**	3.431	3.555	3.426	3.423	4.414	1	26
**SCPT_SPEC**	0.027	0.027	0.027	0.027	0.036	0.767	1
**ListSort_Unadj**	9.007	9.16	9.001	9.065	11.257	80.79	144.5
**MMSE_Score**	0.786	0.779	0.774	0.793	1.012	23	30
**PSQI_Score**	2.109	2.103	2.111	2.107	2.737	0	19
**Endurance_Unadj**	9.108	9.441	9.051	9.09	11.925	80.78	145.17
**GaitSpeed_Unadj**	0.157	0.158	0.158	0.158	0.196	0.79	2
**Dexterity_Unadj**	8.558	8.585	8.5	8.506	10.616	85.15	148.67
**Strength_Unadj**	7.462	8.866	7.876	7.108	11.252	55.29	154.59
**Taste_Unadj**	11.637	11.723	11.538	11.612	14.232	56.35	134.65
**Emotion_Task_Face_Acc**	2.184	2.172	2.183	2.207	3.669	43.333	100
**Language_Task_Math_Avg_Difficulty_Level**	0.553	0.556	0.553	0.552	0.613	1.143	3.857
**Language_Task_Story_Avg_Difficulty_Level**	1.159	1.193	1.152	1.156	1.47	3.786	12.571
**Relational_Task_Acc**	10.282	10.41	10.211	10.139	12.623	35.417	100
**WM_Task_Acc**	6.905	7.054	6.883	6.875	9.135	25	100
**NEOFAC_A**	4.589	4.652	4.575	4.589	5.799	10	48
**NEOFAC_O**	4.937	4.943	4.925	4.922	6.186	10	47
**NEOFAC_C**	4.66	4.677	4.637	4.662	5.858	11	48
**NEOFAC_E**	4.809	4.789	4.779	4.824	5.948	10	47
**AngAggr_Unadj**	7.4	7.49	7.302	7.366	8.811	43.4	83.1
**FearAffect_Unadj**	6.104	6.07	6.076	6.147	7.943	32.9	84.9
**Sadness_Unadj**	6.185	6.165	6.164	6.239	7.845	34.2	76.9
**LifeSatisf_Unadj**	7.383	7.392	7.376	7.41	9.178	23.7	74.6
**MeanPurp_Unadj**	6.885	6.864	6.843	6.908	8.681	29.4	71.6
**Loneliness_Unadj**	6.818	6.768	6.789	6.854	8.554	37.6	82.9
**PercStress_Unadj**	7.214	7.2	7.214	7.262	9.19	22.4	80.5
**SelfEff_Unadj**	6.788	6.81	6.763	6.805	8.299	24.1	68.4

Table S11. Prediction error (mean absolute error or MAE) for each non-brain-imaging phenotype in the HCP-YA dataset. Results are shown for K = 100 and are averaged across 100 random samplings of the 100 participants. The standard deviation, minimum and maximum of the ground truth phenotypic values across participants are also reported for reference.

	MAE				Phenotypic values		
**HCP-Aging phenotype**	**Elastic net**	**Transfer learning**	**MM finetune**	**MM stacking**	**Standard deviation**	**Minimum**	**Maximum**
**LifeSatisf**	7.614	7.68	7.553	7.509	9.872	16	76
**MeanPurp**	6.742	6.795	6.719	6.818	8.615	27	71
**PosAffect**	5.652	5.67	5.563	5.659	7.637	23	71
**Sadness**	7.758	7.791	7.739	7.787	9.865	30	84
**FearAffect**	7.21	7.336	7.206	7.22	9.547	27	92
**FearSomat**	7.406	7.41	7.385	7.373	8.635	38	85
**AngAffect**	7.38	7.415	7.335	7.348	9.397	27	82
**AngHostil**	8.194	8.333	8.196	8.134	9.714	36	78
**AngAggr**	6.427	6.494	6.428	6.385	7.581	42	77
**ER40ANG**	0.913	0.937	0.904	0.899	1.176	2	8
**ER40FEAR**	1.088	1.103	1.095	1.073	1.387	0	8
**ER40NOE**	1.177	1.188	1.17	1.185	1.504	0	8
**ER40SAD**	1.163	1.182	1.161	1.159	1.466	0	8
**EmotSupp**	7.77	7.782	7.733	7.783	9.509	13	62
**InstruSupp**	8.705	8.747	8.67	8.736	10.612	21	63
**Friendship**	7.518	7.55	7.473	7.548	9.489	16	67
**Loneliness**	7.876	7.964	7.863	7.854	9.927	37	85
**PercReject**	8.144	8.124	8.089	8.178	9.825	36	88
**PercHostil**	6.613	6.702	6.617	6.656	8.456	33	75
**PercStress**	7.165	7.228	7.152	7.126	9.115	23	78
**SelfEff**	7.113	7.114	7.084	7.09	8.656	28	68
**NEOFAC_A**	4.49	4.483	4.45	4.482	5.546	15	48
**NEOFAC_O**	4.737	4.763	4.721	4.705	5.924	12	45
**NEOFAC_C**	4.996	5.025	4.971	5.032	6.293	12	48
**NEOFAC_N**	5.983	6.063	5.97	5.986	7.466	0	42
**NEOFAC_E**	4.803	4.816	4.793	4.813	6.006	9	47
**Endurance**	9.097	10.184	9.319	8.983	13.364	43	140
**GaitSpeed**	0.182	0.186	0.183	0.182	0.245	0.47	2.42
**Strength**	8.152	9.458	9.036	7.9	11.843	71	146
**PicSeq**	11.07	11.748	11.19	10.89	14.559	76	136
**CardSort**	6.228	6.575	6.199	6.216	8.952	52	120
**Flanker**	5.815	6.199	5.773	5.888	8.761	57	117
**ReadEng**	5.607	5.684	5.566	5.514	7.137	85	155
**PicVocab**	9.24	9.522	9.211	9.053	12.013	68	139
**ProcSpeed**	13.014	14.281	12.867	12.836	18.836	50	220
**DDisc_AUC_40K**	0.237	0.243	0.239	0.235	0.278	0.016	0.984
**ListSort**	8.726	9.083	8.696	8.689	11.662	55	136
**raw_vat**	4.782	5.131	4.671	4.679	6.947	61	95
**raw_pain**	2.894	2.933	2.902	2.896	3.268	5	29
**winright_ncorr**	3.948	4.233	3.816	3.749	6.147	0	30
**psqi_total**	9.312	9.394	9.272	9.361	12.532	44.444	163.636
**tmta_raw**	8.514	9.159	8.242	8.224	12.989	12.16	132.56
**tmtb_raw**	30.108	31.092	29.405	29.691	60.092	18.54	427.15
**pea_ravlt_sd_tc**	10.597	11.202	10.617	10.41	14.145	16	99
**moca_total**	2.012	2.092	2.044	2.008	2.553	18	31

Table S12. Prediction error (mean absolute error or MAE) for each non-brain-imaging phenotype in the HCP-Aging dataset. Results are shown for K = 100 and are averaged across 100

random samplings of the 100 participants. The standard deviation, minimum and maximum of the ground truth phenotypic values across participants are also reported for reference.

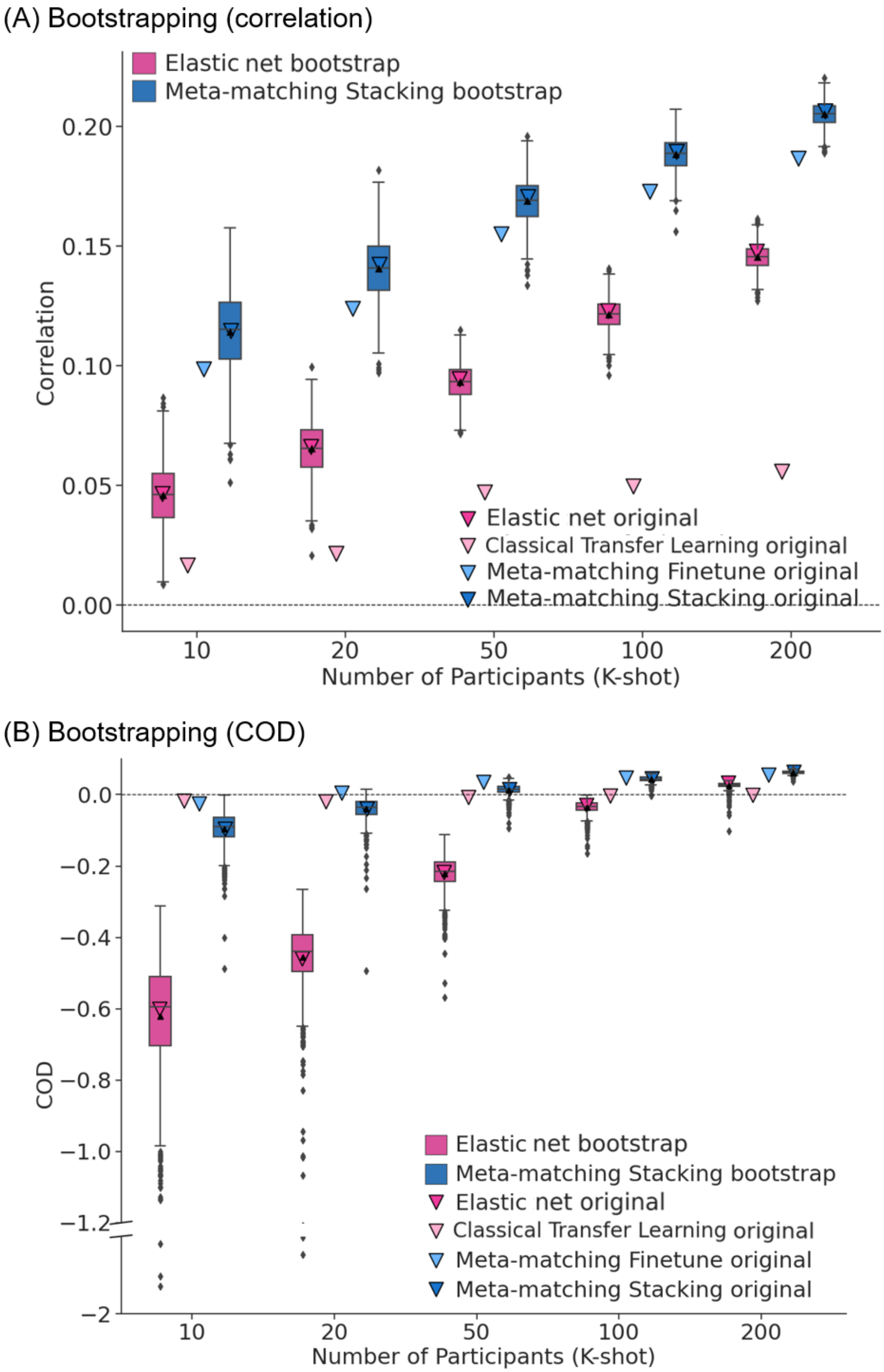
(A) Bootstrapping (correlation)
Elastic net bootstrap
Meta-matching Stacking bootstrap
Correlation
0.20
0.15
0.10
0.05
0.00
Elastic net original
Classical Transfer Learning original
Meta-matching Finetune original
Meta-matching Stacking original
10
20
50
100
200
Number of Participants (K-shot)
(B) Bootstrapping (COD)
COD
0.0
−0.2
−0.4
−0.6
−0.8
−1.0
−1.2
−2
Elastic net bootstrap
Meta-matching Stacking bootstrap
Elastic net original
Classical Transfer Learning original
Meta-matching Finetune original
Meta-matching Stacking original
10
20
50
100
200
Number of Participants (K-shot)

Figure S1. Meta-matching outperformed elastic net and classical transfer learning within UK Biobank bootstrapping experiment. (A) Phenotypic prediction performance (Pearson's correlation) (averaged across 34 meta-test phenotypes) on bootstrapped samples in the meta-test set of UK Biobank. X-axis is the number of participants in the meta-test set of UK Biobank used to train an elastic net baseline or adapt the pretrained model from the meta-training set of UK Biobank. Boxplot shows the distribution of performance over 1,000 repetitions of bootstrapped K participants. Inverted triangles represent the average prediction performance (Pearson's correlation) on 100 repetitions of K participants. (B) Same analysis for COD.

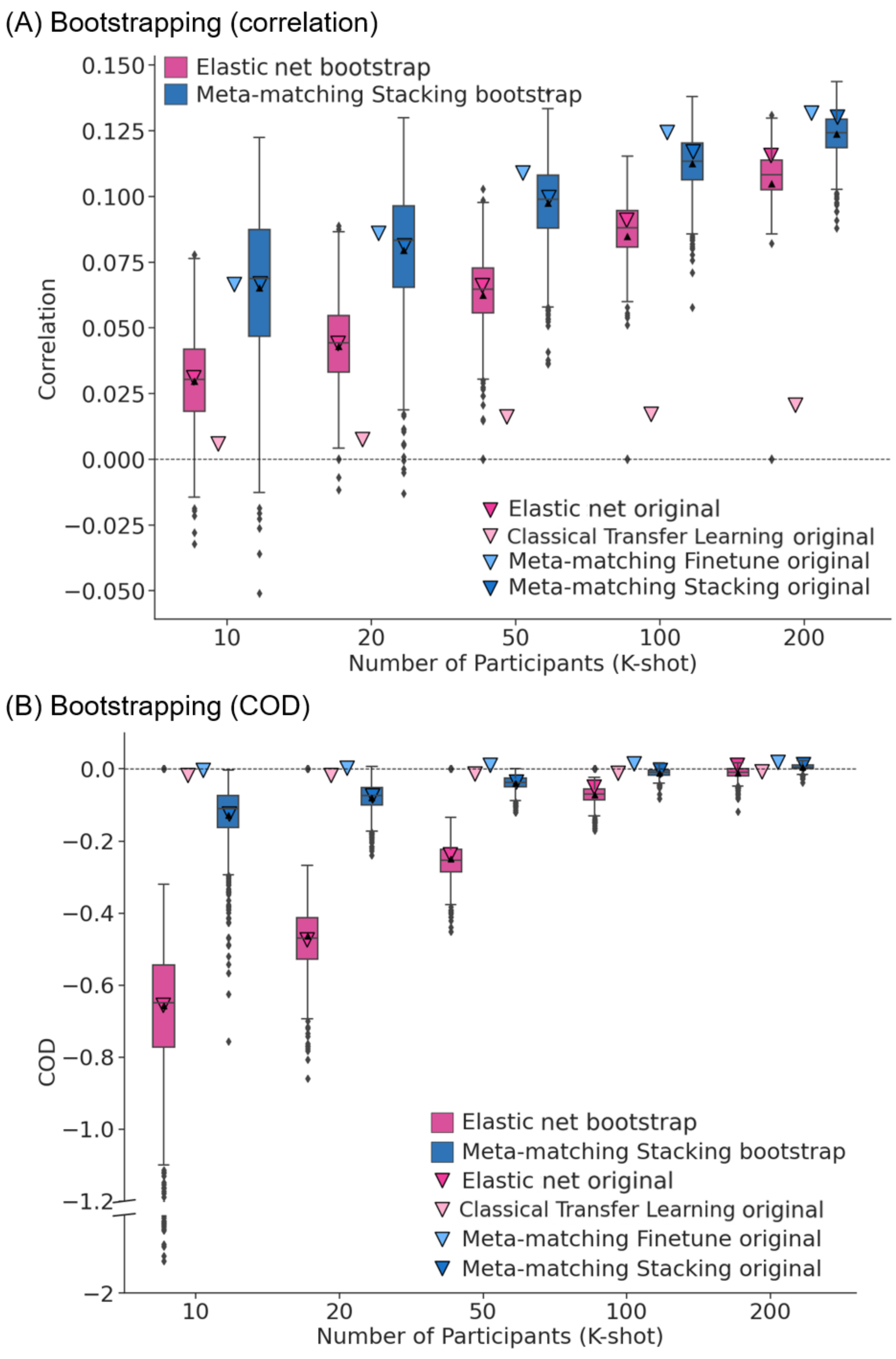
(A) Bootstrapping (correlation)
Elastic net bootstrap
Meta-matching Stacking bootstrap
Elastic net original
Classical Transfer Learning original
Meta-matching Finetune original
Meta-matching Stacking original
Correlation
0.150
0.125
0.100
0.075
0.050
0.025
0.000
−0.025
−0.050
10
20
50
100
200
Number of Participants (K-shot)
(B) Bootstrapping (COD)
COD
0.0
−0.2
−0.4
−0.6
−0.8
−1.0
−1.2
−2
Elastic net bootstrap
Meta-matching Stacking bootstrap
Elastic net original
Classical Transfer Learning original
Meta-matching Finetune original
Meta-matching Stacking original
10
20
50
100
200
Number of Participants (K-shot)

Figure S2. Meta-matching outperformed elastic net and classical transfer learning for HCP-YA bootstrapping experiment. (A) Phenotypic prediction performance (Pearson's correlation) (averaged across 35 meta-test phenotypes) on bootstrapped samples in the HCP-YA dataset. X-axis is the number of participants in the HCP-YA dataset used to train an elastic net baseline or adapt the pretrained model from the meta-training source dataset (UK Biobank). Each boxplot shows the distribution of performance over 1,000 repetitions of bootstrapped K participants. (B) Same analysis for COD.

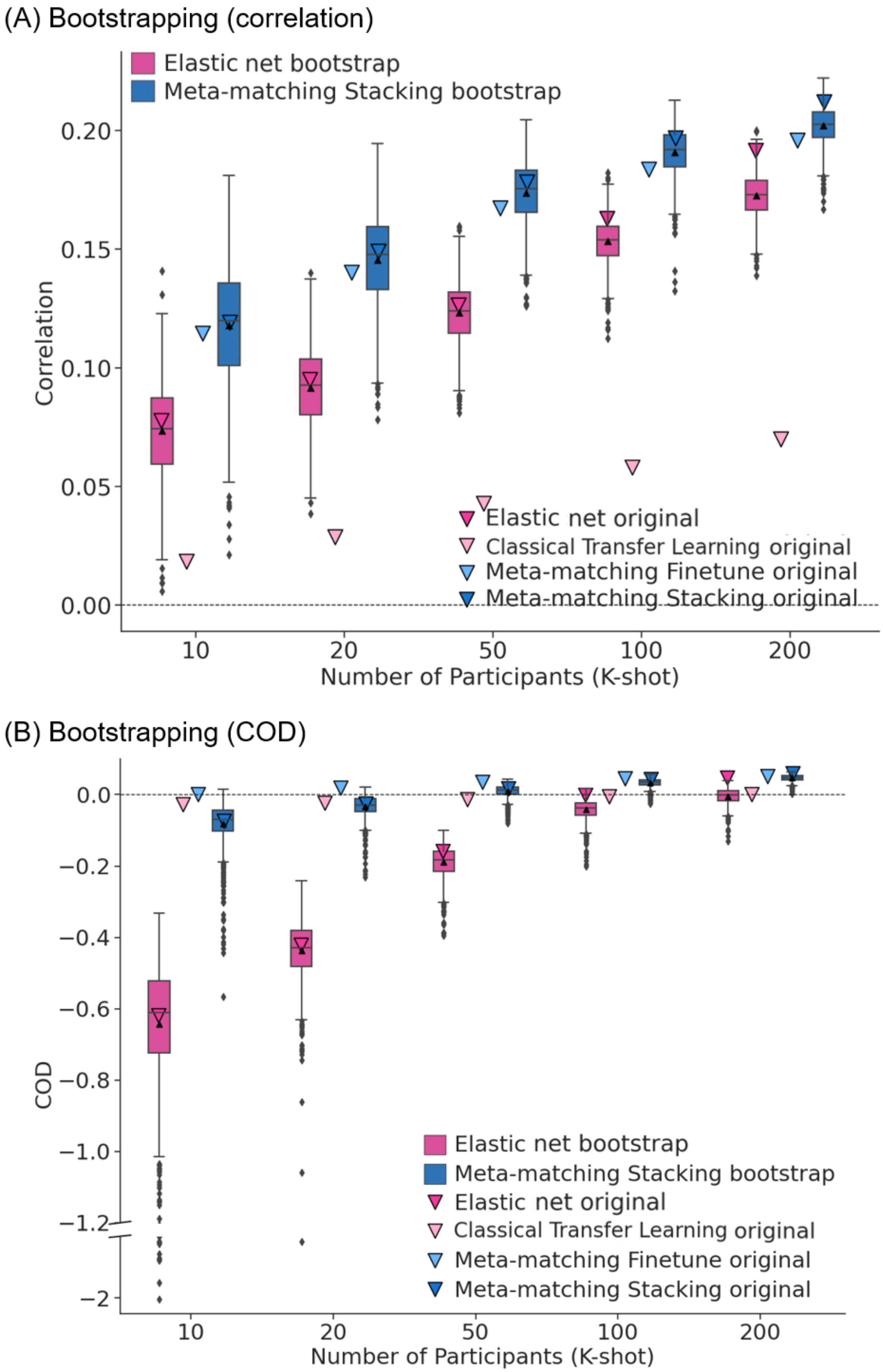
(A) Bootstrapping (correlation)
Elastic net bootstrap
Meta-matching Stacking bootstrap
Elastic net original
Classical Transfer Learning original
Meta-matching Finetune original
Meta-matching Stacking original
Correlation
0.20
0.15
0.10
0.05
0.00
10
20
50
100
200
Number of Participants (K-shot)
(B) Bootstrapping (COD)
Elastic net bootstrap
Meta-matching Stacking bootstrap
Elastic net original
Classical Transfer Learning original
Meta-matching Finetune original
Meta-matching Stacking original
COD
0.0
−0.2
−0.4
−0.6
−0.8
−1.0
−1.2
−2
10
20
50
100
200
Number of Participants (K-shot)

Figure S3. Meta-matching outperformed elastic net and classical transfer learning for HCP-Aging bootstrapping experiment. (A) Phenotypic prediction performance (Pearson's correlation) (averaged across 45 meta-test phenotypes) on bootstrapped samples in the HCP-Aging dataset. X-axis is the number of participants in the HCP-Aging dataset used to train an elastic net baseline or adapt the pretrained model from the meta-training source dataset (UK Biobank). Each boxplot shows the distribution of performance over 1,000 repetitions of bootstrapped K participants. (B) Same analysis for COD.

References

Feilong, M., Guntupalli, J. S., & Haxby, J. V. (2021). The neural basis of intelligence in fine-grained cortical topographies. *Elife*, *10*, e64058.
